# Development and reporting of artificial intelligence in osteoporosis management

**DOI:** 10.1093/jbmr/zjae131

**Published:** 2024-08-20

**Authors:** Guillaume Gatineau, Enisa Shevroja, Colin Vendrami, Elena Gonzalez-Rodriguez, William D Leslie, Olivier Lamy, Didier Hans

**Affiliations:** Interdisciplinary Center of Bone Diseases, Rheumatology Unit, Bone and Joint Department, Lausanne University Hospital and University of Lausanne, Av. Pierre-Decker 4, 1011 Lausanne, Switzerland; Interdisciplinary Center of Bone Diseases, Rheumatology Unit, Bone and Joint Department, Lausanne University Hospital and University of Lausanne, Av. Pierre-Decker 4, 1011 Lausanne, Switzerland; Interdisciplinary Center of Bone Diseases, Rheumatology Unit, Bone and Joint Department, Lausanne University Hospital and University of Lausanne, Av. Pierre-Decker 4, 1011 Lausanne, Switzerland; Interdisciplinary Center of Bone Diseases, Rheumatology Unit, Bone and Joint Department, Lausanne University Hospital and University of Lausanne, Av. Pierre-Decker 4, 1011 Lausanne, Switzerland; Department of Medicine, University of Manitoba, Winnipeg, MB R3T 2N2, Canada; Internal Medicine Unit, Internal Medicine Department, Lausanne University Hospital and University of Lausanne, 1005 Lausanne, Switzerland; Interdisciplinary Center of Bone Diseases, Rheumatology Unit, Bone and Joint Department, Lausanne University Hospital and University of Lausanne, Av. Pierre-Decker 4, 1011 Lausanne, Switzerland

**Keywords:** Analysis/quantitation of bone, osteoporosis, orthopaedics, fracture risk assessment, screening

## Abstract

An abundance of medical data and enhanced computational power have led to a surge in artificial intelligence (AI) applications. Published studies involving AI in bone and osteoporosis research have increased exponentially, raising the need for transparent model development and reporting strategies. This review offers a comprehensive overview and systematic quality assessment of AI articles in osteoporosis while highlighting recent advancements. A systematic search in the PubMed database, from December 17, 2020 to February 1, 2023 was conducted to identify AI articles that relate to osteoporosis. The quality assessment of the studies relied on the systematic evaluation of 12 quality items derived from the minimum information about clinical artificial intelligence modeling checklist. The systematic search yielded 97 articles that fell into 5 areas; bone properties assessment (11 articles), osteoporosis classification (26 articles), fracture detection/classification (25 articles), risk prediction (24 articles), and bone segmentation (11 articles). The average quality score for each study area was 8.9 (range: 7–11) for bone properties assessment, 7.8 (range: 5–11) for osteoporosis classification, 8.4 (range: 7–11) for fracture detection, 7.6 (range: 4–11) for risk prediction, and 9.0 (range: 6–11) for bone segmentation. A sixth area, AI-driven clinical decision support, identified the studies from the 5 preceding areas that aimed to improve clinician efficiency, diagnostic accuracy, and patient outcomes through AI-driven models and opportunistic screening by automating or assisting with specific clinical tasks in complex scenarios. The current work highlights disparities in study quality and a lack of standardized reporting practices. Despite these limitations, a wide range of models and examination strategies have shown promising outcomes to aid in the earlier diagnosis and improve clinical decision-making. Through careful consideration of sources of bias in model performance assessment, the field can build confidence in AI-based approaches, ultimately leading to improved clinical workflows and patient outcomes.

## Graphical Abstract

**Graphical Abstract:**
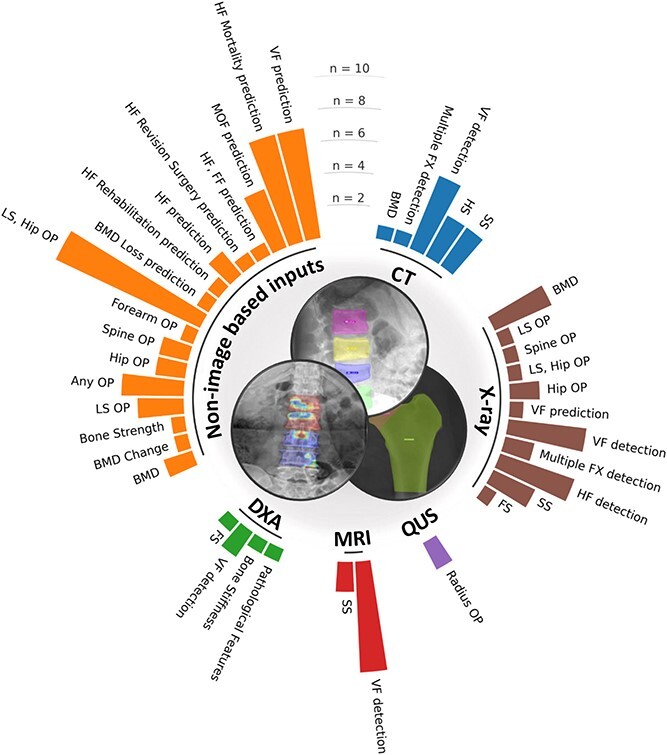

## Introduction

Artificial intelligence (AI) and its subfields, machine learning (ML) and deep-learning (DL), are revolutionizing many domains by developing algorithmic tools that mimic human reasoning and behaviors through the identification of high-dimensional patterns in data.^1^ This involves training complex models and architectures for task-specific questions. AI is being applied to complex medical scenarios and multifactorial conditions. However, AI models may produce unreliable or misleading results, prompting researchers to develop explainable AI (XAI) methods. XAI aims to enhance the interpretability and explainability of the models, making the decision-making process more transparent and accessible to humans.^2^

Osteoporosis is a systemic skeletal disorder characterized by compromised bone strength, increasing the risk of fractures. It is an increasingly prevalent condition that affects up to 1 in 2 women and 1 in 5 men after the age of 50.^3^^,^^4^ Fractures lead to significant health, societal, and economic burdens.^4^ DXA is the reference standard for diagnosing osteoporosis, assessing BMD. Determining fracture risk is crucial in osteoporosis prevention and treatment. FRAX® is the most widely used tool for quantifying fracture risk, by integrating clinical risk factors and DXA-derived bone parameters.^5^ However, FRAX® remains an imperfect tool, and there is a need for more accessible and accurate tools for identifying patients with elevated risk of osteoporosis and/or fracture. The availability of vast amounts of medical data and improved computing power has facilitated an exponential increase in studies using ML and DL methods in bone research using clinical and imaging data.^6^^,^^7^

## Objectives

This review aims to summarize recent advancements in AI for osteoporosis management, report state-of-the-art AI methods, and evaluate key quality items associated with these methods. It is a logical update of the qualitative review by Smets et al. published in 2020,^7^ serving a broad audience from clinicians to researchers.

## Materials and methods

### Literature search strategy

A relevant search strategy to fracture and osteoporosis was created with a Medical Library expert from University of Lausanne (C.J). A systematic search was performed in PubMed from December 17, 2020 (end date of the systematic search performed by Smets et al.) to February 1, 2023. The search strategy is provided in the Supplementary Material (section I).

### Study selection

All identified records were extracted and imported into Rayyan, where duplicates were removed and the titles and abstracts were screened.^8^ Full-text records were then retrieved after this initial selection. Inclusion criteria were: original study, written in English, use of AI methods, and gold standard approaches for osteoporosis management, including confirmed densitometric assessment for osteoporosis or fragility fractures of the forearm, hip, spine, or humerus.

### Study qualitative analysis

The minimum information about clinical artificial intelligence modeling^7^ checklist was used for qualitative assessment. This 6-part checklist includes subsections to avoid common misuses or pitfalls, and rigorously report study design, data and optimization, model performance, model examination, and reproducibility. A simplified version of the checklist was created from the 12 most relevant items to cover the 6 main quality assessment parts (Supplementary Material section 2, Box S1).

Each article was scored with a maximum of 12 points, and methodological details summarizing key study characteristics were retrieved: country (population), task, input data modality, amount of data for model development, amount of data for external validation (EV), number of inputs, model architecture, cross-validation (CV) or train/validation/test split, evaluation metrics, best results, and quality score.

## Results

### Search strategy

**Figure 1:**
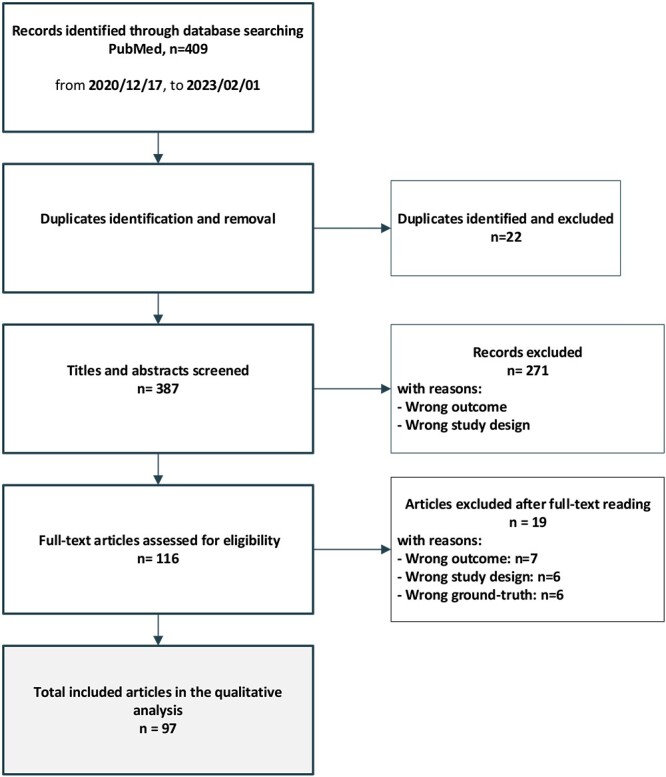
Literature search strategy and workflow.

The search identified 409 records from PubMed. A total of 97 articles meeting inclusion criteria were included in this qualitative review. The workflow and results of the search are summarized in Figure 1.

## General characteristics of the studies

**Figure 2:**
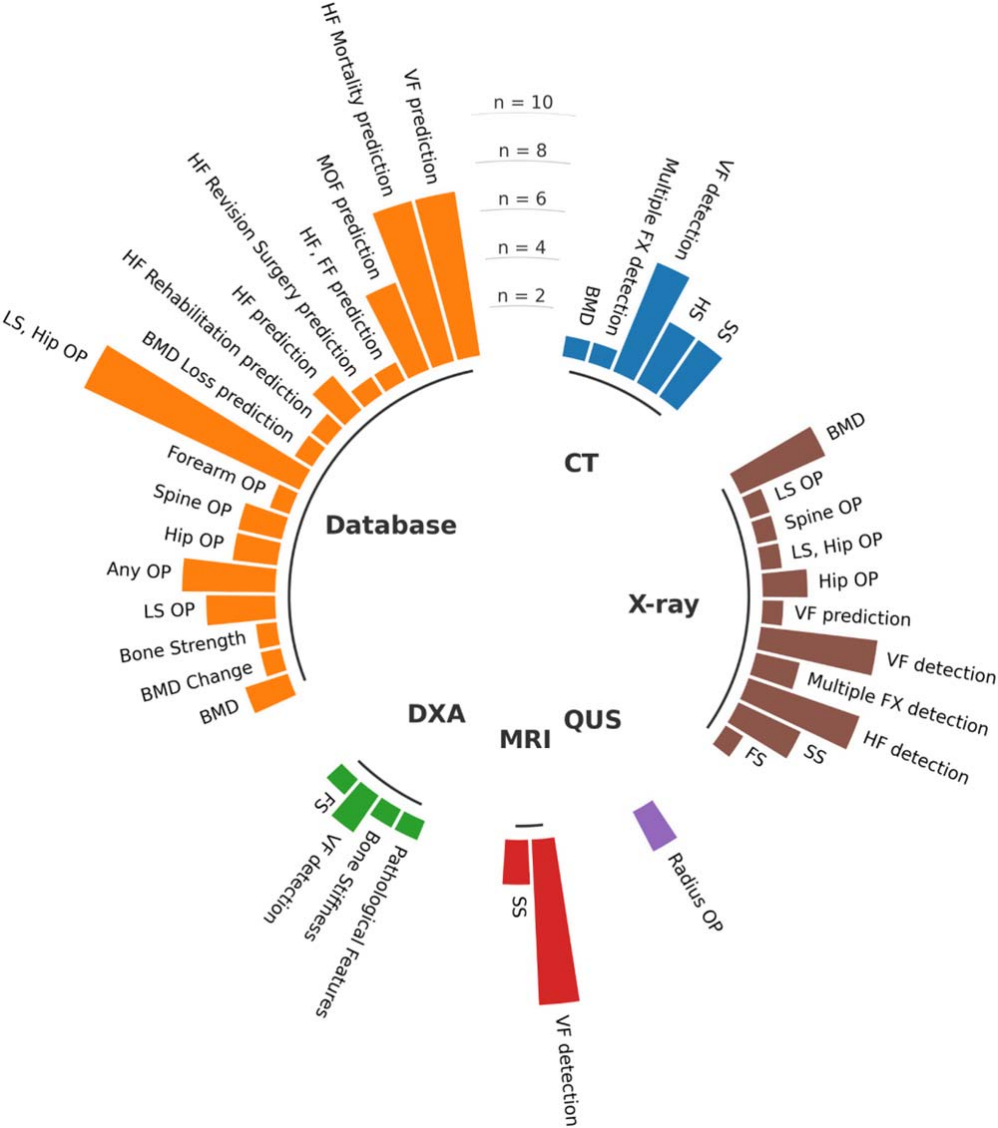
Distribution of study tasks per input modalities included in this qualitative review. FX, fracture; FF, forearm fracture; FS, forearm segmentation; HF, hip fracture; HS, hip segmentation; MOF, major osteoporotic fracture; OP, osteoporosis; SS, spine segmentation; VF, vertebral fracture. As an example, studies that used X-ray input images investigated BMD measurements, LS OP classification, spine OP classification, hip OP classification, LS or hip OP classification, VF prediction, VF detection, multiple anatomical sites FX detection, HF detection, SS and forearm segmentation (FS).

The 97 included articles fell within 5 broad areas: bone properties assessment (11 articles), osteoporosis classification (26 articles), fracture detection/classification (25 articles), risk prediction (24 articles), and bone segmentation (11 articles). Tables 1–5 provide a comprehensive summary of the general characteristics, methodological details, main results, and overall quality score for each of the 5 study areas. Most studies were performed in Asia (68%), with China (27%), South Korea (13%), and the USA (13%) being the most represented countries. ML input data modalities included databases (49%), X-rays (27%), CT (15%), MRI (9%), DXA images (5%), and QUS (2%). To note, database input modalities represent any form of input data that are not in visual image format, such as structured information combining qualitative or quantitative features. Most database studies included DXA-derived measurements such as BMD. Thus, DXA was a data source in more than 5% of studies. Similarly, CT was a data source in more than 15% of the studies, as some database studies include Hounsfield Units (HU) derived from CT scans. Figure 2 presents the distribution of all study tasks per input modality for the 97 included articles.

**Table 1:** General characteristics, methodological details, main results, and overall quality score for bone properties studies.

**Reference**	**Country (population)**	**Task**	**Modality**	**Data amount = *n* (slices)**	**Data amount external validation**	**Inputs (no.)**	**Model**	**Train/validation/test**	**K-fold cross validation**	**Evaluation metrics**	**Best result**	**Quality score (max 12)**
**Dai et al.^9^**	China	BMD	Database	245		1218	Stacking Linreg, RF, XGB, **stacking ensemble**	80% train, 20% test	10-fold	Adjusted R2, RMSE, MAE	R2 = 0.83	9
**Ho et al.^10^**	Taiwan	BMD	X-ray, database	5027		IMG^3^	ResNet-18	80% train, 20% test	5-fold	Pearson’s correlation coefficient, accuracy, sensitivity, specificity	R2 = 0.85, Accuracy = 0.88	10
**Hsieh et al.^11^**	Taiwan	BMD	X-ray, database	36 279	5406	IMG patches	VGG-11, VGG-16, **ResNet-18**, ResNet-34, ensemble	50% train, 50% test	4-fold	Pearson’s correlation coefficient, R2, RMSE, calibration slope, calibration bias	R2 = 0.9	11
**Kang et al.^12^**	South Korea	BMD	CT	547		IMG patches	U-net and custom ResNet	83% train, 17% test	10-fold	Pearson’s correlation coefficient, MAPE, accuracy, specificity, sensitivity, F1-score	R2 = 0.90	7
**Min et al.^13^**	South Korea	BMD	Database	736		45	Linreg, **MLP**	80% train, 20% test	10-fold	MSE, Pearson’s correlation coefficient	R2 = 0.78	7
**Nguyen et al.^14^**	South Korea	BMD	X-ray, database	660		IMG	Custom VGG ensemble	5-fold 80% train, 20% test	5-fold	Pearson’s correlation coefficient, MAE	R2 = 0.81	9
**Nissinen et al.^15^**	Finland	Pathological features	DXA	2949	574	IMG	VGG-16, VGG-19, Inception-V3, DenseNet-121, **custom**	10-fold train/validation	10-fold	Accuracy, confusion matrix, sensitivity, specificity, AUC	AUC = 0.94	11
**Sato et al.^16^**	Japan	BMD	X-ray	17 899		IMG patches	ResNet-50	70% train, 10% validation, 20% test		Pearson’s correlation coefficient, MAE, BA, calibration slope, calibration bias	R2 = 0.75	7
**Tanphiriyakun et al.^17^**	Thailand	BMD change	Database	13 562		225	**RF**, XGB, Logreg, SVM, NB, MLP, KNN	90% train, 10% test	3-fold	Accuracy, precision, recall, F1-Score, AUC	AUC = 0.7	8
**Xiao et al.^18^**	USA	Bone stiffness	DXA	522		IMG^1–3^^–^^6–9^	CNN	80% train, 20% test		Pearson’s correlation coefficient, norm error	R2 = 0.97	9
**Zhang et al.^19^**	China	Bone strength	Database	80		46	SVR	68% train, 32% test	10-fold	MSE, R2, mean bias, SD bias	MSE = 0.014, R2 = 0.93	10

Abbreviations: IMG, image; SVR, support vector regression; CNN, convolutional neural network; Linreg, linear regression; Logreg, logistic regression; RF, random forests; XGB, extreme gradient boosting; SVM, support vector machines; NB, Naïve Bayes; MLP, multilayer perceptron; R2, coefficient of determination; RMSE, root-mean-square error; AUC = area under the curve of the receiver operating characteristic; MAE, mean absolute error; MAPE, mean absolute percentage error; MSE, mean squared error; BA, Bland–Altman. If several models were evaluated for a given task, the best performing is highlighted in bold.

**Table 2:** General characteristics, methodological details, main results, and overall quality score for osteoporosis classification studies.

**Reference**	**Country (population)**	**Task**	**Modality**	**Data amount (%OP)**	**Data amount external validation (%OP)**	**Inputs (no.)**	**Model**	**CV train / validation / test**	**K-fold cross validation**	**Evaluation metrics**	**Best result**	**Quality score (max 12)**
**Biamonte et al.^20^**	Italy	Spine OP	Database	240 (24%)		20	SVM	75% train, 25% test		Accuracy, sensitivity, specificity, AUC	AUC: 0.79	6
**Bui et al.^21^**	Vietnam	LS + Hip OP	Database	1951 (28,9%)		15	Logreg, SVM, **RF**, MLP	80% train, 20% test	5-fold	AUC, precision, recall, F1-score	AUC: 0.85	9
**Chen et al.^22^**	China	Radius OP	QUS	114 (29%)		4	**CNN**, RF	75% train, 12% validation, 13% test	5-fold	Accuracy, sensitivity, specificity, Kappa, AUROC, AUC	AUC: 0.80	9
**Chen et al.^23^**	Taiwan	LS OP	Database	197 (51%)	396 (1.3%)	44	ResNet50 + SVM	5-fold CV	5-fold	AUC, accuracy, sensitivity, specificity, PPV, NPV	AUC: 0.98	7
**Erjiang et al.^24^**	Ireland	LS + Hip OP	Database	13 577 (18.1%)		30	CatBoost, **XGB**, MLP, bagged flexible discriminant analysis (BFDa), RF, Logreg, SVM	Stratified, 80% train, 20% validation		AUC	AUC: 0.83	9
**Fasihi et al.^25^**	Iran	LS + Hip OP	Database	1224 (18%)		19	ET, RF, KNN, SVM, **GB**, extra trees (ET), AdaBoost, MLP	80% train, 20% test		AUC, accuracy, precision, sensitivity, specificity, F-score	AUC: 0.95	5
**Huang et al.^26^**	China	LS + Hip OP	Database	172 (47.7 %)		826	GNB, RF, Logreg, SVM, **GBM**, XGB	60% train, 40% test	5-fold	AUC, sensitivity, specificity, accuracy	AUC: 0.86	8
**Jang et al.^27^**	South Korea	Spine OP	X-ray	13 026 (33%)	1089 (29%)	IMG	Inception-v3	70% train, 10% validation, 20% test		AUC, accuracy, sensitivity, specificity	AUC: 0.88	10
**Jang et al.^28^**	South Korea	Hip OP	X-ray	1001 (50.3%)	117 (73.5%)	IMG	VGG16 + NLNN	Stratified, 80% train, 10% validation, 10% test		Accuracy, sensitivity, specificity, AUC, PPV, NPV	AUC: 0.87	8
**Kwon et al.^29^**	South Korea	LS + Hip OP	Database	1431 (57%)		1151	RF, **AdaBoost**, GBM	80% train, 20% test	5-fold	AUC	AUC: 0.91	8
**Liu et al.^30^**	China	Spine OP	Database	246		28	**Logreg,** SVM, MLP, RF, XGBoost, Stacking	80% train, 20% test	10-fold	AUC	AUC: 0.96	7
**Luo et al.^31^**	China	Radius OP	QUS	274 (34%)		4	CNN	70% train, 15% validation, 15% test	5-fold	Accuracy, sensitivity, specificity, PPV, NPV, Kappa	Accuracy: 0.83	9
**Mao et al.^32^**	China	LS OP	X-ray, database	5652 (33%)	628 (34%)	IMG (+3)	DenseNet	80% train, 10% validation, 10% test		AUC, sensitivity, specificity, PPV, NPV	AUC: 0.95	10
**Ou Yang et al.^33^**	Taiwan	Any OP	Database	5982 (7%)		19	MLP, **SVM**, RF, KNN, Logreg	65% train, 15% validation, 20% test		AUC, sensitivity, specificity, Youden’s index	AUC: 0.84	8
**Park et al.^34^**	South Korea	LS + Hip OP	Database	3309 (9.2%)		20	Logreg, **XGB**, MLP	70% train, 30% validation	10-fold	AUROC, AUPRC, sensitivity, specificity	AUC: 0.79	10
**Sebro et al.^35^**	USA	LS + Hip OP	Database	253 (74%)		10	RF, XGBoost, NB, **SVM**	70% train, 30% test	10-fold	AUC, accuracy, sensitivity, specificity, PPV, NPV	AUC: 0.756	7
**Sebro et al.^36^**	USA	LS + Hip OP	Database	394 (78%)		15	Logreg, LASSO, **SVM**	70% train, 30% test	10-fold	AUC, accuracy, sensitivity, specificity, PPV, NPV	AUC: 0.89	7
**Sebro et al.^37^**	USA	LS + Hip OP	Database	364 (22.3%)		15	Lasso, elastic net, ridge regression, **SVM with RBF**	80% train, 20% test	10-fold	AUC, accuracy, sensitivity, specificity, PPV, NPV	AUC: 0.86	7
**Sebro et al.^38^**	USA	Forearm OP	Database	196 (27.6%)		24	**SVM**, RF	49% train, 51% test	10-fold	AUC, sensitivity, specificity, accuracy, PPV, NPV	AUC: 0.82	7
**Shen et al.^39^**	USA	Any OP	Database	211 (23.2%)		178	ZeroR, OneR, J48 tree, **RF**, KNN, Logreg, SVM, NB	80% train, 20% test	10-fold	Accuracy, sensitivity, specificity, AUC	AUC: 0.84	7
**Suh et al.^40^**	South Korea	Hip OP	Database	8680 (3.1%) and 8274 (3.4%)		89 and 162	SVM, DT, ET, LGBM, Logreg, KNN, **MLP**	5-fold CV	5-fold	AUC	AUC: 0.92	7
**Sukegawa et al.^41^**	Japan	LS + Hip OP	X-ray, database	778 (30%)		IMG + 3	EfficientNet-b0, EfficientNet-b3, EfficientNet-b7, ResNet-18, ResNet-50, ResNet-152, **Ensemble**	80% train, 20% test	5-fold	Accuracy, AUC, precision, recall, specificity, F1 score	AUC: 0.92	11
**Wang et al.^42^**	China	Any OP	Database	1419 (32%)		18	**MLP**, deep belief network (DBN), SVM, GA-DT	70% train, 30% test		Accuracy, Sensitivity, Specificity, AUC	AUC: 0.90	6
**Widyaningrum et al.^43^**	Indonesia	Any OP	Database	102 (49%)		15	DT, NB, **MLP**	60% train, 40% test		Accuracy, sensitivity, specificity	Accuracy: 0.90	6
**Yamamoto et al.^44^**	Japan	Hip OP	X-ray, database	1699 (53%)		IMG + 3	ResNet-18, ResNet-34, ResNet-50, ResNet-101, ResNet-152, **ensemble**	stratified,75% train, 25% test	4-fold	Accuracy, precision, recall, specificity, F1 Score, AUC	AUC: 0.89	9
**Yang et al.^45^**	China	LS OP	Database	1046 (28%)		41	Logreg	NA	NA	AUC, sensitivity, specificity	AUC: 0.97	5

Abbreviations: OP, osteoporosis; IMG, image; Logreg, logistic regression; XGB, extreme gradient boosting; MLP, multilayer perceptron; RF, random forests; SVM, support vector machines; CNN, convolutional neural network; GBM, Gradient Boosting Machine; KNN, k-nearest neighbors; GNB, Gaussian naïve Bayes; LASSO, least absolute shrinkage and selection operator; NB, Naïve Bayes; DT, decision tree; ET, extra trees, LGBM, light gradient boosting machine; RBF, radial basis functions; GA-DT, genetic algorithm - decision tree; ET, extra trees; GB, gradient boosting; AUC, area under the curve of the receiver operating characteristic; NPV, negative predictive value; PPV, positive predictive value; NA, not available. If several models were evaluated for a given task, the best performing is highlighted in bold.

**Table 3:** General characteristics, methodological details, main results, and overall quality score for fracture detection/classification studies.

**Reference**	**Country (population)**	**Task**	**Modality**	**Data amount = *n* (slices) (% fractures)**	**Data amount external validation (% fractures)**	**Inputs (no.)**	**Model**	**CV train/validation/test**	**K-fold cross validation**	**Evaluation metrics**	**Best result**	**Quality score (max 12)**
**Bae et al.^46^**	South Korea	HF	X-ray	4189 (26%)	2099 (25%)	IMG	ResNet-18	80% train, 10% validation, 10% test		Sensitivity, specificity, AUC, Youden index	AUC 0.98	9
**Chen et al.^47^**	China	VF	X-ray	3754 (50%)		IMG	ResNeSt-50 18	67% train, 33% test	5-fold	Accuracy, sensitivity, AUC	AUC: 0.80	11
**Cheng et al.^48^**	Taiwan	HF	X-ray	7092 (61%)		IMG	DenseNet-169	75% train, 25% test	5-fold	Accuracy, sensitivity, specificity, AUC, PPV, NPV	AUC: 0.97	10
**Chou et al.^49^**	Taiwan	VF	X-ray	7459 (15%)	1281 (8%)	IMG patches	YOLOv3, ResNet-34, DenseNet121, DenseNet201, **ensemble**	60% train, 20% validation, 20% test		Accuracy, sensitivity, specificity	Accuracy: 0.92	8
**Del Lama et al.^50^**	Brazil	VF	MRI	189 (53%)		IMG patches + features^51^	VGG16, InceptionV3, Xception, **hybrid**	89% train, 11% test	10-fold	Precision, recall, f1-score, support, specificity, sensitivity, balanced accuracy	Balanced accuracy: 0.88	9
**Dong et al.^52^**	USA	VF	X-ray	100 409 (1.2%)		IMG patches^3^	GoogLeNet	76.5% train, 8.5% validation, 15% test		AUC, AUC-PR	AUC: 0.99	10
**Germann et al.^53^**	Switzerland	VF	MRI	1000 (23.8%)		IMG	U-Net	79% train, 9.8% validation, 9.8% test. 1.4% development		Accuracy, sensitivity, specificity, dice, ICC, Kappa	Accuracy: 0.96	10
**Guermazi et al.^54^**	USA	Multiple	X-ray	60 170 (NR)	480 (50%)	IMG	Detectron2	70% train, 10% validation, 20% test		Sensitivity, specificity, AUC	AUC: 0.93	9
**Inoue et al.^55^**	Japan	Multiple	CT	200 = 7664 (5.8%)		IMG	Faster R-CNN	90% train, 10% test		Sensitivity, precision, F1-score	Sensitivity: 0.79	7
**Li et al.^56^**	China	VF	CT	433 (68%)		IMG	ResNet-50	10-fold CV	10-fold	Sensitivity, specificity, accuracy	Accuracy: 0.85	7
**Li et al.^57^**	Taiwan	VF	CT, MRI	941 (17%)	52 (27%)	IMG	ResNet-34, ResNet-50, DenseNet121, DenseNet160, DenseNet201, **ensemble**	60% train, 20% validation, 20% test		Accuracy, sensitivity, specificity, AUC, Cohen’s Kappa	Accuracy: 0.89	8
**Monchka et al.^58^**	Canada	VF	DXA	31 152 (15.5%)		IMG	Inception-ResNet-v2	71% train, 1% validation, 28% test		Balanced accuracy, sensitivity, specificity, PPV, NPV, F1-score	Balanced accuracy: 0.95	8
**Monchka et al.^59^**	Canada	VF	DXA	12 742 (17%)		IMG	Inception-ResNet-v2, DenseNet, **ensemble**	60% train, 10% validation, 30% test		Accuracy, balanced accuracy, sensitivity, specificity, PPV, NPV, F1-score, AUC	AUC: 0.95	10
**Murphy et al.^60^**	UK	HF	X-ray	3659 (56%)		IMG	GoogLeNet	60% train, 20% validation, 20% test		Accuracy, Cohen’s Kappa, AUC, F1-score	Accuracy: 0.92	10
**Ozkaya et al.^61^**	Turkey	VF	X-ray	390 (49%)		IMG	ResNet-50	52% train, 22% validation, 26% test		Sensitivity, specificity, AUC	AUC 0.84	5
**Rosenberg et al.^62^**	Switzerland	VF	CT, MRI	222 = 630 (48%)		IMG patches	VGG16, **ResNet18**	90% train, 10% test	10-fold	Accuracy, sensitivity, specificity, NPV, AUC	Accuracy: 0.88	7
**Sato et al.^63^**	Japan	HF	X-ray	10 484 (50%)		IMG^3^	EfficientNet-B4	80% train, 10% validation, 10% test		Accuracy, sensitivity, specificity, F1-score, AUC	Accuracy: 0.96	9
**Twinprai et al.^64^**	Thailand	HF	X-ray	1000 (50%)		IMG	Yolov4-tiny	90% train, 10% test		Accuracy, precision, sensitivity, specificity, F1-score	Accuracy: 0.95	8
**Xu et al.^65^**	China	VF	X-ray	1460 = 2031 (100%)	444 = 578	IMG	ResNet-18	80% train, 20% test		Accuracy, sensitivity, specificity, AUC, precision, F1-score, PPV, NPV	Accuracy: 0.83	11
**Yabu et al.^66^**	Japan	VF	MRI	1624 (60%)		IMG	VGG-16, VGG-19, DenseNet-121, DenseNet-169, DenseNet-201, InceptionResNet-V2, Inception-V3, ResNet-50, Xception, **ensemble**	60% train, 40% test		Accuracy, sensitivity, specificity, AUC	AUC: 0.95	5
**Yadav et al.^67^**	India	Multiple	X-ray	34 000 augmented (50%)		IMG	AlexNet, VGG16, ResNeXt, MobileNetV2, **SFNet**	80% train, 20% test		Precision, recall, f1-score, accuracy	Accuracy: 0.99	7
**Yeh et al.^68^**	Taiwan	VF	MRI	190 (26%)		IMG^3^	ResNet-50	10-fold CV	10-fold	Accuracy	Accuracy: 0.92	9
**Yoda et al.^69^**	China	VF	MRI	112 = 697 (48%)		IMG	Xception	5-fold CV	5-fold	Accuracy, sensitivity, specificity, AUC	AUC: 0.98	8
**Zakharov et al.^70^**	Russia	VF	CT	100 = 3565 (21%)	300 and 100 (50%)	IMG	Custom CNN	5-fold CV	5-fold	Accuracy, precision, recall	AUC: 0.95	8
**Zhang et al.^71^**	China	VF	CT	1217 (96%)		IMG	U-GCN, 3DResNet	70% train, 10% validation, 20% test		Accuracy, balanced accuracy, sensitivity, specificity, AUC	Accuracy: 0.98 (detection), balanced accuracy: 0.80 (classification)	6

Abbreviations: HF, hip fracture; VF, vertebral fracture; MRI, magnetic resonance imaging; DXA, dual-energy X-ray absorptiometry; CT, computed tomography; IMG, image; CNN, convolutional neural network; AUC, area under the curve of the receiver operating characteristic; AUC-PR, area under the precision-recall curve; PPV, positive predictive value; NPV, negative predictive value; ICC, intra-class correlation coefficient. If several models were evaluated for a given task, the best performing is highlighted in bold.

**Table 4:** General characteristics, methodological details, main results, and overall quality score for risk prediction studies.

**Reference**	**Country (population)**	**Task (prediction time)**	**Modality**	**Data amount (% cases)**	**Data amount external validation (% cases)**	**Inputs (no.)**	**Model**	**CV train / validation / test**	**K-fold cross validation**	**Evaluation metrics**	**Best result**	**Quality score (max 12)**
**Cary et al.^72^**	USA	HF Mortality (30 d, 1 yr)	Database	17 140 (15%)		15	Logreg, **MLP**	10-fold CV	10-fold	Accuracy, AUC, precision, slope	AUC: 0.76	8
**Chen et al.^73^**	China	VF (5 yr)	Database	1603 (8%)		147	Logreg, SVM, DT, KNN, RF, ERT, GBDT, AdaBoost, CatBoost, XGB, MLP, **hybrid**	80% train, 20% test	NR	Accuracy, precision, recall, F1-score, AUC	Accuracy: 0.90	9
**Chen et al.^74^**	China	MOF (6 yr)	Database	487 (NA)		22	**RF**, MLP, SVM, XGB, DT	70% train, 30% test	10-fold	AUC, DCA, CIC	AUC: 0.87	9
**Cheng et al.^75^**	Taiwan	BMD Loss (6 yr)	Database	23 497 (14%)		17	Logreg, **XGB**, RF, SVM	80% train, 20% test	10-fold	Sensitivity, specificity, AUC, accuracy, precision, f1-score	AUC: 0.75	7
**Coco Martín et al.^76^**	Spain	MOF (> 1 yr)	Database	993 (28%)		25	**Logreg**, MLP	70% train, 30% test		Accuracy	Accuracy: 0.96	7
**De Vries et al.^77^**	Netherlands	MOF (3 yr, 5 yr)	Database	7578 (11%)		46	**Coxreg**, RSF, MLP	10-fold CV	10-fold	C-index	C-Index: 0.70	11
**DeBaun et al.^78^**	USA	HF Mortality (30 d)	Database	19 835 (1051)		43	**MLP**, naive Bayes, Logreg	80% train, 20% test		AUC	AUC: 0.92	4
**Du et al.^79^**	China	HF	Database	120 (NA)		13	R2U-Net and SVM, RF, GBDT, AdaBoost, **MLP**, XGB	80% train, 20% test		Accuracy, specificity, recall, precision	Accuracy: 0.96	5
**Forssten et al.^80^**	Sweden	HF Mortality (1 yr)	Database	124 707 (17%)		25	**Logref**, SVM, RF, NB	80%train, 20% test	5-fold	Accuracy, sensitivity, specificity, AUC	AUC: 0.74	11
**Galassi et al.^81^**	Spain	HF	Database	137 (65%)		38	Logreg, SVM, DT, **RF**	70% train, 30% test		Sensitivity, specificity, accuracy	Accuracy: 0.87	6
**Harris et al.^82^**	USA	HF Mortality (30 d)	Database	82 168 (5%)		46	LASSO	10-fold	10-fold	Accuracy, C-Index	Accuracy: 0.76	8
**Kitcharanant et al.^83^**	Thailand	HF Mortality (1 yr)	Database	492 (13%)		15	GB, **RF**, MLP, Logreg, NB, SVM, KNN	70% train, 30% test		Accuracy, sensitivity, specificity, AUC, PPV, NPV	AUC: 0.99	10
**Klemt et al.^84^**	USA	HF Revision Surgery (> 2 yr)	Database	350 (5.2%)		NR	**MLP**, RF, KNN, PLR	80% train, 20% test	5-fold	AUC, intercept, calibration, Brier score	AUC: 0.81	6
**Kong et al.^85^**	South Korea	VF	Database, X-ray	1595 (7.5%)		IMG patches (+7)	HRNet + ResNet and **DeepSurv**, Coxreg	89% train, 11% test	5-fold	AUC, sensitivity, specificity, PPV, NPV, C-Index	C-Index: 0.61	11
**Lei et al.^86^**	China	HF Mortality	Database	391 (13.8%)	165 (10.9%)	27	RF, GBM, DT, **XGB**	67% train, 33% test	10-fold	AUC, accuracy, sensitivity, specificity, Youden Index, intercept, calibration slope	AUC: 0.71	7
**Lu et al.^87^**	UK	MOF	Database	345 (28%)		359	**Logreg**, RF	80% train, 20% test	5-fold	AUC, sensitivity, specificity	AUC: 0.90	7
**Ma et al.^88^**	China	VF	Database	529 (10.6%)		27	DT, **RF**, SVM, GBM, MLP, RDA, Logreg	75% train, 25% test	10-fold	AUC, Kappa, sensitivity, specificity	AUC: 0.94	9
**Oosterhoff et al.^89^**	Netherlands	HF Mortality (90 d, 2 yr)	Database	2478 (9.1% and 23.5%)		14	**SGB,** RF, SVM, MLP, **PLR**	80% train, 20% test	10-fold	AUC, intercept, calibration, Brier score	AUC: 0.74 (90 d), AUC: 0.70 (2 yr)	9
**Poullain et al.^90^**	France	VF	Database	60 (50%)		16	RF, CART	k-fold	k-fold	Sensitivity, specificity, AUC	AUC: 0.92	5
**Shimizu et al.^91^**	Japan	HF, FF (> 2 yr)	Database	6590 (4.4%)		10	**LightGBM,** ANN	75% train, 25% test		AUC	AUC: 0.75	4
**Shtar et al.^92^**	Israel	HF Rehabilitation (8 yr)	Database	1896 (14%)		18	Linreg, Logreg, AdaBoost, CatBoost, ExtraTrees, KNN, RF, SVM, XGB, **ensemble**	NR	10-fold	AUC, R2	AUC: 0.86	10
**Takahashi et al.^93^**	Japan	VF (Nonunion)	Database	153 (17%)		17	Logreg, DT, XGB, **RF**	70% train, 30% test	5-fold	AUC, accuracy	AUC: 0.86	9
**Ulivieri et al.^94^**	Italy	VF (9 yr)	Database	174 (69)		9	MLP	70% train, 30% test		Sensitivity, specificity, AUC, accuracy	AUC: 0.82	5
**Ulivieri et al.^95^**	Italy	VF (3 yr)	Database	172 (54%)		26	MLP	NR		Sensitivity, specificity, accuracy, AUC	Accuracy: 0.79	6

Abbreviations: VF, vertebral fracture; HF, hip fracture; FF, forearm fracture; MOF, major osteoporotic fracture; BMD, bone mineral density; NA, non assessable; MLP, multilayer perceptron; Logreg, logistic regression; GB, gradient boosting; RF, random forests; NB, Naïve Bayes; SVM, support vector machines; KNN, k-nearest neighbors; SGB, stochastic gradient boosting; PLR, Elastic-Net Penalized Logistic Regression; DT, decision tree; Linreg, linear regression; XGB, extreme gradient boosting; Coxreg, Cox regression; RSF, random survival forests; ERT, extremely randomized trees; GBDT, gradient boosted decision trees; GBM, gradient boosting machines; RDA, regularized discriminant analysis; CART, classification and regression tree; LASSO, least absolute shrinkage and selection operator; AUC, area under the curve of the receiver operating characteristic; PPV, positive predictive value; NPV, negative predictive value; C-Index, concordance index; R2, coefficient of determination; DCA, decision curve analysis; CIC, clinical impact curve. If several models were evaluated for a given task, the best performing is highlighted in bold.

**Table 5:** General characteristics, methodological details, main results, and overall quality score for bone segmentation studies.

**Reference**	**Country (population)**	**Task**	**Modality**	**Data amount**	**Data amount external validation**	**Inputs (no.)**	**Model**	**CV train / validation / test**	**K-fold cross validation**	**Evaluation metrics**	**Best result**	**Quality score (max 12)**
**Cheng et al.^96^**	China	SS	CT	15	15	IMG^10^	Custom U-Net	66.6% train, 33.3% test		Dice coefficient, location error, detection rate, IoU, Hausdorff distance, pixel accuracy	Dice coefficient: 0.95	10
**Deng et al.^97^**	China	HS	CT	100		IMG	U-Net	85% train, 15% test	10-fold	Dice coefficient, average surface distance, sensitivity, specificity	Dice coefficient: 0.98	10
**Kim et al.^98^**	South Korea	SS	X-ray	797		IMG	U-Net, **hybrid**	80% train, 20% test		Dice coefficient, precision, sensitivity, specificity, area error, Hausdorff distance	Dice coefficient: 0.92	10
**Kim et al.^99^**	South Korea	SS	X-ray	339		IMG	U-Net, R2U-Net, SegNet, E-Net, **dilated recurrent residual U-Net**	80% train, 20% test	5-fold	Sensitivity, specificity, accuracy, dice coefficient	Dice coefficient: 0.93	7
**Park et al.^100^**	South Korea	SS	CT	467	102	IMG	U-Net	80% train, 20% test		Dice coefficient	Dice coefficient: 0.93	11
**Suri et al.^101^**	USA	SS	X-ray, CT, MRI	6975		IMG	Custom CNN	5-fold	5-fold	Accuracy, IoU, dice coefficient	Dice coefficient: 0.95	10
**Wang et al.^102^**	China	HS	CT	50		IMG	U-Net	66% train, 20% validation, 14% test	5-fold	Dice coefficient, precision, sensitivity	Dice coefficient: 0.92	8
**Wei et al.^103^**	China	FS	X-ray	1274		IMG	hybrid ResNet+FPN and DeepLabv3	60% train, 20% validation, 20% test		MAP, AUC	AUC: 0.98	10
**Yang et al.^104^**	China	FS	DXA	720		IMG^2^	U-Net Resblock	83% train-validation, 17% test	5-fold	Dice coefficient, Jaccard index	Dice coefficient: 0.99	10
**Yang et al.^105^**	China	HS	CT	160		IMG	DenseUnet and Mask R-CNN	75% train, 25% test		Accuracy, dice coefficient	Accuracy: 0.89, dice: 0.90	6
**Zhao et al.^106^**	China	SS	MRI	222	25	IMG	U-Net	70% train, 30% test		Dice coefficient	Dice coefficient: 0.912	7

Abbreviations: FS, forearm segmentation; SS, spine segmentation; HS, hip segmentation; IMG, image; CNN, convolutional neural network; IoU, intersection over union; MAP, mean average precision; AUC, area under the curve of the receiver operating characteristic. If several models were evaluated for a given task, the best performing is highlighted in bold.

## Qualitative analysis of the studies

**Figure 3:**
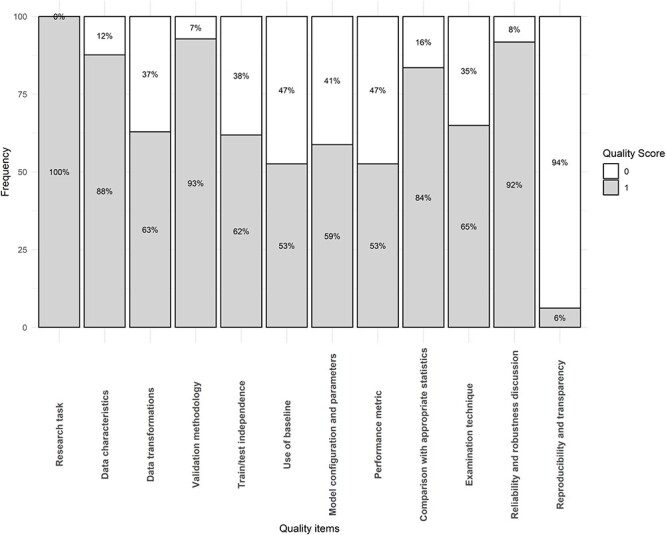
Quality scores per item for all included studies (*n* = 97).

The percentage of studies fulfilling each quality item is represented in Figure 3. The average quality score for each study area was 8.9 (range: 7–11) for bone properties assessment, 7.8 (range: 5–11) for osteoporosis classification, 8.4 (range: 7–11) for fracture detection, 7.6 (range: 4–11) for risk prediction, and 9.0 (range: 6–11) for bone segmentation.

The 3 most fulfilled items were: research task definition (100%), validation methodology (93%), and reliability and robustness discussion (92%). The least fulfilled items were reproducibility and transparency (6%), use of a baseline model for performance comparison (53%), and justification of performance metrics (53%).

For deeper interest in AI subfields and model selection, the reader can refer to the Supplementary Material, Section 3. Supplementary Material, Box S1, gives an overview of the key ML/DL development, evaluation, and reporting steps. Concepts of data stratification, CV, classification tasks, regression tasks, segmentation tasks, and their respective performance metrics are provided in Tables S1–S3.

## Studies on bone properties

Eleven studies evaluated AI models for bone properties assessment, including BMD prediction,^9–14^ BMD change over time,^17^ pathological features detection,^15^ bone stiffness assessment,^18^ and bone strength prediction.^19^ The objective of these studies was to develop opportunistic screening tools to assess and predict bone health parameters to intervene at an earlier stage and improve the prognosis of individuals at high risk of osteoporotic fracture. Study details are presented in Table 1, and their individual quality scores are specified in Table S4.

AI models for BMD prediction used diverse approaches and input modalities. BMD prediction from spine, hip, or chest radiographs was investigated with convolutional neural networks (CNNs).^10^^,^^11^^,^^16^ Hip BMD prediction from X-ray was performed as a regression task for the first time.^10^^,^^11^ The ResNet-18 architecture was selected to predict hip BMD from pelvis X-rays. The selection of this model was motivated by its lighter architecture compared with larger neural networks that are better suited to multi-class classification tasks.^10^ Two image pre-processing approaches for hip BMD prediction compared a ROI with and without surrounding soft tissues. The model utilizing a ROI with surrounding soft tissues had the best correlation with DXA BMD (r = 0.84 ± 0.02 vs r = 0.81 ± 0.03). Gaussian occlusion sensitivity (GOS) enabled heat-map visualizations showing model activations in both the femur and adjacent soft tissues. Hsieh and colleagues advanced CNN applications for BMD prediction with a multi-stage approach.^11^ First, a deep adaptive graph CNN was used for precise anatomical landmark detection and ROI extraction of the LS vertebrae and hip, serving as an opportunistic vertebral fracture (VF) detection tool with 93.2% sensitivity and 91.5% specificity on independent cases. Then, PelviXNet CNN architectures identified cases with structural bone changes, including vertebral compression fractures (VCFs), hip fractures (HFs), and surgical implants. Finally, ResNet and VGG architectures, each augmented with transfer learning, achieved BMD prediction. ResNet-18 and ResNet-34 provided the best results for hip BMD (r = 0.917), while VGG-16 performed best for spine BMD (r = 0.900). The robust performance across stages and successful EV highlighted the multi-stage approach’s potential for opportunistic BMD screening. Other pre-processing approaches for hip BMD prediction involved Sobel gradient filtering applied to 3 different Singh ROIs of 160 x 160 pixels.^14^ The Sobel gradient filtering enhanced trabecular patterns, significantly improving the DXA BMD correlation from 0.308 to 0.723. An ensemble of VGG-nets, each trained on one specific Singh ROI, aggregated the BMD predictions, improving the correlation (r = 0.807).

BMD prediction was also achieved from CT using CNNs with image slices^12^ or ML regression models using image-derived features such as HU and/or radiomics.^9^^,^^13^^,^^19^ BMD prediction from CT image slices using a ResNet-101v2 architecture showed a correlation of 0.905 with DXA BMD when including surrounding soft tissues, compared to 0.878 when these tissues were removed, corroborating previous findings for hip BMD prediction from X-rays.^10^

BMD change and response to treatment were investigated using multiple ML algorithms with various input features, including demographic data, diagnoses, laboratory results, medications, and initial BMD measurements.^17^ Nissinen et al. developed and evaluated different CNN architectures for detecting pathological features from DXA images, including severe scoliosis and unreliable BMD measurements due to structural abnormalities.^15^ Xiao et al. used DXA images to predict the apparent stiffness tensor of cadaveric trabecular bone cubes.^18^ Their model was trained with micro-CT-based finite element simulations as ground truth. Finally, Zhang et al. evaluated a support vector machine (SVM) regressor to predict femoral strength in elderly men from QCT-based finite element analysis.^19^

Six studies (54%) involved baseline models for comparison. Incorporating clinical variables such as age, height, and weight into CNN architectures improved hip BMD prediction from X-rays, increasing the correlation from 0.766 to 0.807.^14^ The performance of an image-based CNN showed equivalent performance with histomorphometry and bone/volume/fraction tensor parameter-based regression models for predicting the apparent stiffness tensor of trabecular bone cubes.^18^ Zhang and colleagues compared the performance of support vector regression models using different sets of input features and dimensionality reduction for predicting femoral strength from QCT.^19^ They obtained the best results by reducing the dimensionality from 46 radiomics to 12 components with principal component analysis (PCA),^107^ keeping more than 95% of the explained variance. Hsieh et al. compared FRAX performance using DL-predicted BMD from X-ray or DXA BMD, finding no significant differences.^11^ Nissinen et al. compared custom CNN architectures with classical CNN classifiers for predicting scoliosis and unreliability in BMD measurements from LS DXA scans.^15^ Automation of hyperparameter tuning involved random search and Hyperband. Their model outperformed radiologists in scoliosis detection (94.1% vs 92.5%) and in terms of image unreliability (82.4% vs 78.8%). Finally, Dai et al. compared the performance of common ML algorithms against a 2-tier stacking ensemble model to predict spine BMD from CT images. The stacking ensemble model yielded superior performances with correlation and calibration bias with DXA BMD of 0.932 and −0.01 ± 0.14 mg/cm^2^, respectively.^9^

Image-based features or variable contributions were investigated in 6 studies (54%). Heat maps were generated using GOS maps,^10^ gradient-weighted class activation mapping (Grad-CAM),^12^^,^^15^ or vanilla gradient descent.^15^ Innovative strategies enabled heat-map visualizations from a regressor CNN instead of a classifier.^12^ Feature engineering strategies included PCA and Least Absolute Shrinkage and Selection Operator (LASSO)^108^ for dimensionality reduction,^9^^,^^19^ and Shapley Additive explanations (SHAP)^109^ for ranking variable importance and contribution.^17^ The LASSO model efficiently reduced the dimensionality of CT radiomics from 1218 features to 11 for predicting spine BMD.

## Studies on osteoporosis classification

Twenty-six studies on osteoporosis classification were included; their characteristics are summarized in Table 2, and quality assessment in Table S5. The objective of these studies was to furnish novel screening tools for opportunistic osteoporosis identification. The ground-truth for osteoporosis diagnosis was DXA, using LS,^20–27^^,^^30^^,^^34–37^^,^^41^^,^^45^ hip,^21^^,^^24–26^^,^^28^^,^^34–36^^,^^41^^,^^44^ forearm,^22^^,^^31^^,^^38^ or the minimum BMD T-score of these regions.^33^^,^^42^^,^^43^ Input modalities included databases,^20^^,^^21^^,^^23–26^^,^^30^^,^^33–37^^,^^40–45^ X-ray images,^32^^,^^41^^,^^44^ QUS signals,^22^^,^^31^ or the combination of images and patient characteristics.^32^^,^^41^^,^^44^ Image-based osteoporosis classification involved CNNs in every study with common architectures,^23^^,^^27^^,^^28^^,^^32^ multi-stage approaches,^31^ or ensemble models.^22^^,^^41^^,^^44^ Studies without image input used ML models with numerical features derived from CT,^20^^,^^23^^,^^26^^,^^35^^,^^37^^,^^38^^,^^45^ X-ray image features,^43^ population-based data, and electronic medical records^20^^,^^21^^,^^24^^,^^25^^,^^29^^,^^33^^,^^34^^,^  ^39^^,^^40^^,^^42^ or a combination of CT radiomics and clinical attributes.^30^ CT bone images and ROIs were obtained from from 3D slicer software,^26^^,^^35–38^ or automatically from AI models including a ResNet CNN to segment vertebral bodies^23^ and the FDA-approved AI-Rad Companion software.^45^ Unsupervised learning techniques including Fuzzy C-means and K-means clustering were used as X-ray pre-processing strategies to cluster pixels into trabecular patterns and predict osteoporosis.^43^ Pre-processing strategies from CT images included the extraction of 12 texture and shape features of vertebral bodies with gray-level co-occurrence matrices and Otsu binarization.^30^ Interestingly, these texture and shape features contributed more to osteoporosis classification than clinical parameters including age, age of menopause, or BMI.

The best performances were seen with boosting algorithms,^22^^,^^24–26^^,^^34^ SVM,^20^^,^^33^^,^^35–38^ RF,^21^^,^^39^ or multilayer perceptron (MLP).^28^^,^^40^^,^^42^^,^^43^ Ten (38%) studies assessed the efficiency of osteoporosis screening models using a baseline model or tool for comparison, such as the osteoporosis risk assessment index, osteoporosis self-assessment tool, osteoporosis self-assessment tool for Asians, osteoporosis index of risk, or simple calculated osteoporosis risk estimation.^21^^,^^24^^,^^31–34^^,^^40^^,^^41^^,^^44^ Consistently, ML models including XGB,^24^^,^^34^ SVM,^33^ MLP,^40^ or RF^21^ outperformed these tools. The synergistic effect of clinical variables with image features extracted from CNNs showed improved osteoporosis classification.^22^^,^^31^^,^^32^^,^^41^^,^^44^

Feature relationships and image regions contributing to osteoporosis classification were explored in several studies.^20^^,^^22^^,^^23^^,^^26–28^^,^^34^^,^^39–41^^,^^45^ Feature engineering strategies were mainly used to gain insights into the most relevant predictors and reduce dimensionality. These strategies included feature importance analysis,^20^^,^^21^^,^^23^^,^^30^^,^^39^ PCA,^22^ Local Interpretable Model-Agnostic Explanations (LIME),^40^ LASSO,^26^^,^^40^ SHAP,^34^ or odds ratios (ORs).^45^ The SHAP and LIME methods were used to visually rank variable importance.^34^^,^^40^ Grad-CAM analysis was used to visualize activated regions from input images.^27^^,^^28^^,^^41^

## Studies on fracture detection/classification

Twenty-five studies investigated fracture detection or classification; their main characteristics are shown in Table 3, and their quality assessment in Table S6. Among them, 20% investigated HF,^46^^,^^48^^,^^60^^,^^63^ 68% VF,^47^^,^^49^^,^^50^^,^^52^^,^^53^^,^^56–59^^,^^61^^,^^65^^,^^66^^,^^68–71^ and 12% multiple fracture sites.^54^^,^^64^^,^^67^ Studies used various medical image modalities as input: X-ray (48%),^46–49^^,^^52^^,^^54^^,^  ^60^^,^^63–65^^,^^67^ MRI (28%),^50^^,^^57^^,^^62^^,^^66^^,^^68^^,^^69^ CT (24%),^56^^,^^57^^,^^62^^,^^64^^,^  ^70^^,^^71^ DXA (8%),^58^^,^^59^ and both CT and MRI (8%).^57^^,^^62^

All studies clearly stated the research task and 19 (76%) reported data characteristics.^46–49^^,^^53^^,^^54^^,^^56–60^^,^^63^^,^^64^^,^^66^^,^^68^^,^^69^^,^^71^ Fracture prevalence varied from 1.2% to 100% reflecting different uses between fracture detection and fracture classification.

All fracture detection and classification studies involved CNN architectures. Input images were used in their entirety or pre-processed to extract specific ROIs. ROIs were obtained from manual annotations by specialists,^47^^,^^50^^,^^52^^,^^56^^,^^61–63^^,^^65^^,^^69^ or automatically extracted using DL models including You Only Look Once (YOLO),^49^^,^^57^^,^^64^ U-net architectures,^53^^,^^71^ 3D U-Net for CT,^70^ or a custom CNN.^60^ Studies that used the whole image as input mainly focused on detecting the presence of a fracture (yes/no classification) rather than classifying its type.^46^^,^^48^^,^^54^^,^^55^^,^^58^^,^^59^ In this context, heatmaps or bounding boxes highlighted regions suggesting fracture(s).^46^^,^^48^^,^^58^^,^^59^

Seventeen studies (68%) used transfer learning to leverage pre-trained models from previous datasets.^47–50^^,^^52^^,^^57^^,^^60–67^^,^^69^ Li et al. demonstrated improved accuracy, from 0.71 to 0.92, when using pre-trained model weights from ImageNet.^57^ Data augmentation techniques were used in 76% of the studies to protect against overfitting.^46–48^^,^^50^^,^^54^^,^^56^^,^^58–60^^,^^62–66^^,^^68^ Techniques included random shifts, rotations, scaling, contrast and brightness enhancement, or mosaic augmentation.^47^ Bae et al. found that model area under the receiver operating characteristic curve (AUC) increased from 0.88 to 0.99 when using data augmentation.^46^ However, Del Lama et al. reported a decrease in most metrics and explained that the augmentations did not accurately reflect characteristics in the original images.^50^

Nineteen studies (76%) involved comparison models or methods.^46^^,^^48–50^^,^^54^^,^^56–61^^,^^63^^,^^65–69^ Among these, 10 (40%) reported equivalent or improved performances using the AI models compared with specialist readings.^53–55^^,^^60^^,^^63–66^^,^^69^^,^^70^ Xu et al. reported that AUCs for VF classification as acute, chronic, or pathological in an external dataset were significantly higher than for the trainee radiologist, similar to the competent radiologist, and only slightly lower than the expert radiologist. All 3 levels of radiological expertise improved with DL-model diagnosis.

Only eleven (44%) studies justified specific metrics used.^46^^,^^47^^,^^54^^,^^60^^,^^62^^,^^64^^,^^65^^,^^68^^,^^70^ For example, Dong et al. prioritized sensitivity, positive predictive value (PPV), and precision-recall curve over AUC due to class imbalance.^52^ Most studies (72%) investigated model decision-making processes using heat-maps,^46^^,^^47^^,^^57–60^^,^^63^ sensitivity analysis,^49^^,^^50^^,^^54^^,^^69^ or error analysis.^52^^,^^53^^,^^68^

### Studies on risk prediction

Twenty-four studies investigated prediction of osteoporosis outcomes such as incident VF,^73^^,^^88^^,^^90^^,^^93–95^ HF or related complications,^72^^,^^78–81^^,^^83^^,^^84^^,^^89^^,^^91^^,^^92^ major osteoporotic fracture (MOF),^76^^,^^77^^,^^87^ or BMD loss.^75^ Study characteristics are summarized in Table 4, and their quality assessments are in Table S7.

Risk prediction studies developed ML and DL models trained from various input data modalities. Studies using non-image-based features trained ML models with retrospective data from large medical registries including demographic, clinical, or laboratory data to predict future events.^72^^,^^74^^,^^75^^,^^78–80^^,^^83^^,^^84^^,^^86^^,^^88^^,^^89^^,^^91^^,^^92^ Other studies developed models that combined clinical parameters and bone measurements or image features. Bone measurements included BMD, bone strain index, or finite element analysis parameters derived from DXA.^74^^,^^77^^,^^81^^,^^94^^,^^95^ Image features involved radiomics from HR-pQCT, CT, and MRI,^87^ GLCM from CT and MRI,^90^ vertebral-level signal changes across time from MRI,^93^ and image features from CNNs.^85^ Combining clinical information with image features showed improved performance for fracture discrimination and prediction.^74^^,^^85^^,^^87^ Notably, the LS X-ray image features extracted from a ResNet CNN showed higher performance than FRAX in terms of C-Index values to predict future VFs (0.612; 95% CI, 0.572-0.656 for the DL model vs 0.547 for FRAX).^85^ Research on optimizing spine X-rays pre-processing for fracture prediction using CNNs revealed that using multiple bounding boxes of L1 to L5 vertebrae including the surrounding soft tissues led to better performance in predicting future fractures. This method outperformed the use of L1 to L5 bounding boxes without the surrounding soft tissues replaced by a black mask, as well as the use of the whole L1-L5 bounding box image with or without a black mask. These results align with Ho et al. and Kang et al. findings^10^^,^^12^ for predicting BMD from pelvis X-ray and suggest that a CNN tends to better analyze bone tissues when the input images include the surrounding tissues.

The best performing models were diverse, and when tested, ensemble voting algorithms or hybrid ML architectures showed improved performances.^73^^,^^92^ Specifically, a hybrid model concatenating a XGB output with an MLP demonstrated significant improvement in all performance metrics to predict future fractures.^73^ Risk prediction studies showed promising results despite heterogeneous quality scores. Selecting and justifying performance metrics for model evaluation were often overlooked, with only 13 (54%) of studies providing justification.^72^^,^^74–76^^,^^80^^,^^83^^,^^85^^,^^86^^,^^88^^,^^89^^,^^92^ CIs were reported in 16 (67%) studies.^72^^,^^77^^,^^80–84^^,^^87–90^^,^^92^^,^^93^

Most risk prediction studies (75%) proposed techniques to explain model behavior and/or visualize informative variable(s).^73–77^^,^^80^^,^^83^^,^^84^^,^^87–90^^,^^92–95^ Methods included semantic connectivity maps from MLP,^94^^,^^95^ SHAP analysis,^83^^,^^92^ feature importance from decision tree architectures,^73^^,^^75^^,^^77^^,^^80^^,^^88^^,^^93^ Boruta algorithm,^110^ LASSO analysis,^82^ and ORs.^76^^,^^87^ MLP and SHAP models provided individual-level risk explanations for the early failure of cementless TH arthroplasty in osteoporotic patients^84^ and mortality in post HF patients.^89^

Only de Vries et al. satisfied the reproducibility and transparency criterion by providing a source code repository.^77^

### Studies on bone segmentation

Eleven studies investigated AI segmentation of various bone regions, including forearm,^103^^,^^104^ hip,^97^^,^^105^ and spine,^96^^,^^98^^,^^100^^,^^101^^,^^106^ from different imaging modalities such as DXA,^104^ MRI,^101^^,^^106^ X-ray,^98^^,^^101^^,^^103^ and CT.^97^^,^^100–102^^,^^105^ Multimodal bone segmentation was investigated by Suri et al. with MRI, CT, and X-ray images.^101^ The main characteristics of bone segmentation studies are provided in Table 5, and their quality assessment is in Table S8. The main goal of bone segmentation is to automatically isolate the bone ROI from surrounding soft tissues or overlapping structures to derive bone measurements such as radiomics, BMD, and VF deformity ratios,^97^^,^^98^^,^^101^^,^^104^^,^^106^ or visualize complex bone regions.^96^^,^^100^^,^^102^^,^^103^^,^^105^ More generally, bone segmentation strategies can be seen as pre-processing strategies to improve the input quality of imaging-based tasks. All studies used CNNs. Nine (82%) considered U-Net based architectures,^96^^,^^97^^,^^99^^,^^100^^,^^102^^,^^104–106^ while others relied on custom architectures.^101^^,^^103^ Hybrid or multi-stage CNN approaches enhanced segmentation of complex bone regions.^96^^,^^98^^,^^103^^,^^105^ These approaches involved separating the tasks of detection and segmentation to achieve more accurate results than segmenting from the whole image. Methods used included a Region Proposal Network (RPN) with precise rotation of the ROIs to reduce redundant background information,^103^ instance segmentation from a Dense U-Net to define the fracture ROI,^105^ and a Pose-Net to predict the coordinates of 5 lumbar vertebrae.^98^

All studies used supervised learning, and the ground truth, or reference mask, was obtained from medical experts in 9 studies (82%),^98^^,^^100–104^^,^^106^ from software in some cases,^97^ or was not reported.^96^ Eight studies (73%) compared performance against baseline models or methods, such as simpler CNN architectures,^98^^,^^103^^,^^104^ software or thresholding methods,^97^^,^^102^ or expert segmentation.^99–101^

To overcome limited numbers of training samples, 8 studies (73%) used data augmentation techniques.^97^^,^^98^^,^^100^^,^^102^^,^^104^^,^^105^

The overall performance of segmentation tasks showed robust results. The primary performance metric used was the Dice coefficient, reported in 10 studies (91%) with an average of 0.93 (SD 0.03; range 0.90-0.99). In 9 studies (82%), model performance was supported with appropriate statistical methods.^97^^,^^98^^,^^100–104^ Strategies to assist in visualizing the predictions were adopted in 8 studies (73%).^96–98^^,^^100^^,^^102^^,^^103^^,^^106^ Three studies (27%) compared model performance with manual expert segmentations and demonstrated no statistical difference.^99–101^

### Studies on clinical decision support

The goal of the studies included in this review was to improve osteoporosis management algorithms and enhance patient outcomes by leveraging advanced ML and DL techniques. This section explores clinical decision support systems for osteoporosis management, utilizing previously evaluated articles from the 5 preceding sections, which aim to improve clinician efficiency, diagnostic accuracy, and patient outcomes through AI-driven models and opportunistic screening. Opportunistic screening addresses the need for cost-effective medical practices by utilizing routinely acquired imaging and clinical data not primarily intended for osteoporosis. Studies demonstrated the effectiveness of using opportunistic images for bone properties assessment and osteoporosis prediction from spine, chest, hip, or panoramic radiographs.^10^^,^^11^^,^^27^^,^^28^^,^^32^^,^^41^ Automated fracture detection tools opportunistically detected fractures in routinely acquired data.^49^^,^^52^^,^^55^^,^^70^^,^^71^ Opportunistic screening helps in earlier diagnosis and fracture risk identification and paves the way for introducing formal diagnostic tests like DXA scans for definitive osteoporosis diagnosis.

AI-driven clinical decision support aims to improve clinician efficiency and diagnostic accuracy by automating or assisting with specific clinical tasks in complex scenarios. These tasks included distinguishing pathological VCFs from those secondary to osteoporosis,^56^^,^^69^^,^^100^ fresh vs old VCFs,^47^^,^^66^ or predicting rehabilitation outcomes and mortality following HFs.^72^^,^^78^^,^^80^^,^^82–84^^,^^89^^,^^92^ Studies exploited ML algorithms to provide individualized risk explanations and further improve patient triage and management.^74^^,^^77^^,^^83^^,^^86^^,^^89^^,^^92^ Moreover, AI promotes consistency in decision-making by mitigating potential human bias and ensuring standardized interpretation of medical data. As fracture types strongly determine the chosen surgical treatment, accurate diagnosis is required to optimize patient outcomes and treatment costs. Studies developed computer-aided diagnosis tools to assist in complex fracture classification.^48^^,^^50^^,^^52^^,^^54^^,^^57^^,^^60^^,^^61^^,^^63^^,^^65^^,^^98^^,^^99^^,^^102^^,^^105^ Importantly, these approaches can empower junior doctors, improving cost-effectiveness, task distribution, and addressing physician shortages. However, translating research into clinical practice requires further development, robust AI models, and seamless integration into established workflows.

Large retrospective electronic medical records were used to develop AI models to optimize treatment plans for reducing BMD loss and fragility fractures.^17^^,^^76^ Tanphiriyakun et al. evaluated an AI model’s ability to predict inadequate treatment response, defined as >3% lumbar BMD loss or >5% femoral BMD loss, using 8981 variables from clinical, laboratory, DXA, and prescription data.^17^ The model’s AUC ranged from 0.61 (KNN) to 0.70 (RF) and provided proof of concept for integration into EMR systems to improve treatment selection and outcomes by identifying novel response predictors. Complementing this research, Martín et al. analyzed anti-osteoporotic treatment responses using logistic regression and neural network models in 993 patients from the OSTEOMED registry.^76^ They found that fracture reduction probabilities were generally independent of sex, age, and comorbidities, though treatments like vitamin D and calcium showed increased efficacy in specific groups. The logistic regression model accurately classified 96% of cases. Both studies highlight the potential of AI-driven approaches to enhance clinical decision support, improving patient care through informed and personalized treatment decisions. Integrating these models into practice could significantly improve outcomes for patients at risk of fragility fractures, enabling more precise and targeted therapeutic interventions.

## Discussion

This review provides a comprehensive summary of the recent AI literature in osteoporosis. Although novel and well-performing ML/DL methods emerged in bone health assessment, the systematic assessment revealed disparities in study quality, highlighting the need for consistent standards in AI development and reporting.

### Quality assessment standards in AI

Among the 97 reviewed studies, only 3 (3%) followed reporting guidelines,^11^^,^^80^^,^^89^ such as the transparent reporting of a multivariable prediction model for individual prognosis or diagnosis (TRIPOD)^111^ and Nature Portfolio (https://www.nature.com/documents/nr-reporting-summary-flat.pdf). Both guidelines are comprehensive for assessing a model. However, these do not cover other important AI methods like data transformations, model optimization strategies, or examination methods that help to identify risks of bias and overfitting. Studies that used reporting guidelines showed better overall quality compared to the others, with an average quality score of 10.3 vs 8.1 (maximum score: 12).

A recent meta-analysis on CNNs for fracture recognition and classification in orthopedics reported a lack of suitable tools to assess the risk of bias in ML.^112^ This meta-analysis relied on the modified Methodologic Index for Non-Randomized Studies^113^ checklist, which is suited for assessing the overall quality of studies with classical predictive statistics, but lacks specificity for AI applications. Kuo et al.^51^ recently conducted a systematic review and meta-analysis of AI for fracture detection and used 2 checklists, the TRIPOD and the Prediction model study Risk Of Bias Assessment Tool.^114^ These checklists were used in combination to provide a general indicator of reporting standards, and to assess potential bias, highlighting the need for AI development and reporting standards that are reflective of the complex methods and unique terminologies.

### Overall quality of the studies

The best scoring studies involved a wide range of sample sizes, from 467 to 124 707. These studies utilized high-quality ground truth (such as osteoporosis, fracture classification or reference bone masks for segmentation tasks), established from gold standard methods or expert consensus. The high quality of the ground truth assessment combined with a proper understanding of the data characteristics facilitated effective model training and relevant feature-outcome associations.

CV strategies provided an assessment of the model’s generalizability and performance, reducing the risk of overfitting. Many studies lacked clear creation of independent training and testing sets.^14^^,^^17^^,^^18^^,^^20^^,^^25^^,^^26^^,^^30^^,^^31^^,^^42^^,^^45^^,^^56^^,^^62–64^^,^^66–69^^,^^72^^,^  ^75–77^^,^^79^^,^^81^^,^^82^^,^^84^^,^^90^^,^^92^^,^^94^^,^^95^^,^^99^^,^^101^^,^^102^^,^^105^ Generating an intermediate validation set or employing CV from the training set can help to prevent overfitting and optimize hyperparameters. Some studies assessed performance on the complete dataset through k-fold CV, meaning that model optimization and evaluation processes were done from the same dataset.^23^^,^^56^^,^^68^^,^^69^^,^^72^^,^^77^^,^^82^^,^^101^ This scenario assumes that the internal dataset is representative of the larger target population and usually demands EV.

### Advances in AI in osteoporosis

AI research in osteoporosis has surged, covering more studies in a shorter period compared to the previous 5 yr assessed by Smets et al. The quality of research has also seen improvement. Utilizing the same scoring employed by Smets et al., this review assessed the overall quality of recent studies. The mean quality score was 8.1, superior to the 7.4 average of the earlier review period. Improvement in quality was supported by a notable increase in model examination strategies (64.8% vs 33.7% from the earlier period) and EVs (16.5% vs 4.5%).

AI tasks within the osteoporosis domain have become increasingly specific, moving beyond general triage to address precise clinical needs. Recent studies focused on diverse applications, including predicting HF complications such as death in hospital, revision surgeries, or rehabilitation outcomes.^72^^,^^78^^,^^80^^,^^82–84^^,^^89^^,^^91^^,^^92^ Combined with relevant feature engineering, AI applications have taken a further step in explaining individualized predictions to enhance therapeutic decisions in specific subgroups of patients with conditions like primary fracture,^83^^,^^86^^,^^89^^,^^92^ rheumatoid arthritis,^74^ osteopenia, and osteoporosis.^77^^,^^95^ AI has aimed at being more precise, supported by innovative multi-factorial AI architectures. For the first time, CNNs were used for predicting BMD as continuous measures from pelvic X-rays, a significant improvement compared with classification-based CNNs.^10^^,^^11^ Strategies for model explainability in DL BMD regression tasks involved the adaptation of classical Grad-CAM to regression tasks (Grad-RAM)^12^ and GOS maps.^10^

Combining clinical information with image-based features or radiomics significantly improved performance compared to standalone clinical or image-based models.^14^^,^^32^^,^^41^^,^^44^^,^^50^^,^^87^ CNN architectures were exploited to combine additional features in the fully connected layers and improve the predictive performance of osteoporosis classifiers from spine, hip, or panoramic radiographs by adding simple covariates.^14^^,^^32^^,^^41^^,^^44^ Notably, combining CNN image features with radiomics, clinical, and histogram information improved the classification of non-traumatic VCFs (fragility) vs malignant (tumors) VCFs from MRI.^50^

In general, researchers have investigated more complex methods to improve pre-processing, explainability, and the overall performance of their models. Including the soft tissues surrounding a bone ROI enhanced BMD prediction and fracture risk assessment from CNNs, highlighting the importance of contextual information.^10^^,^^12^^,^^85^ Unsupervised learning techniques including K-Means and Fuzzy C-Means as pre-processing clustered trabecular bone patterns to enhance osteoporosis prediction.^43^ Similarly, Sobel gradient filtering applied to specific hip ROIs identified relevant trabecular patterns and enhanced BMD prediction.^14^ The continuously evolving YOLO v3 and YOLO v4 architectures showed pre-processing utility to automatically position bounding boxes around bone ROIs in medical images.^49^^,^^57^^,^^64^ Automatic ROI positioning was also achieved through specific segmentation architectures including U-Net, Dense U-net, R2U-Net, SegNet, E-Net, PSPNet, Faster R-CNN, or HRNet. Multi-stage approaches comprising first the ROI extraction and then the bone segmentation by CNN have provided more detailed and accurate segmentation of complex bone structures by reducing information loss and improving the clarity of segmented areas.^103^^,^^105^

### Themes in model performance

Understanding model properties and assumptions is crucial for identifying the best model for a specific application. Researchers developed and evaluated multiple algorithms or incorporated established tools for direct comparison with clinical standards.^9^^,^^11^^,^^14^^,^^15^^,^^18^^,^^21^^,^^24^^,^^31–34^^,^^40^^,^^41^^,^^44^^,^^46^^,^^48–50^^,^^54^^,^  ^56–61^^,^^63^^,^^65–67^^,^^69^^,^^73^^,^^77^^,^^80^^,^^85^^,^^87^^,^^88^^,^^92^^,^^97^^,^^98^^,^^100–104^ The use of statistical approaches in the performance comparison of models has proven valuable in reinforcing and validating findings regarding the best performing models.^9–11^^,^  ^15–18^^,^^20^^,^^22–24^^,^^27^^,^^28^^,^^30–32^^,^^34–42^^,^^44–50^^,^^52–55^^,^^57^^,^^59–61^^,^^63–65^^,^^69^^,^^71^^,^  ^72^^,^^76–94^^,^^97^^,^^98^^,^^100–104^ It is worth noting that 37% of the studies included in this review did not report model performances with CIs, which limits critical evaluation of model performance.

Diversity among best-performing models underscores the importance of considering multiple architectures. Ensemble models demonstrated enhanced performance compared to standalone models, leveraging unique strengths and assigning different weights to input data. Training and optimizing ensemble models require efficient hyperparameter tuning and extensive computational costs. Hyperband optimization, reported in one study,^15^ benefits researchers training large models by speeding up random search through adaptive resource allocation and early stopping of bad runs. DL models, especially CNNs, were extensively used for image-based analysis, with common architectures or customized ensemble and multi-stage models. Transfer learning optimized training processes, particularly with small sample sizes.^16^^,^^41^^,^^44^^,^^47–50^^,^^52^^,^^57^^,^^60–67^^,^^69^

Justifying specific performance metrics for model evaluation was often overlooked.^12^^,^^13^^,^^17^^,^^20^^,^^21^^,^^23^^,^^30^^,^^35–37^^,^^39^^,^^40^^,^  ^42–45^^,^^48–50^^,^^53^^,^^56–59^^,^^61^^,^^63^^,^^66^^,^^69^^,^^73^^,^^78^^,^^79^^,^^81^^,^^87^^,^^90^^,^^91^^,^^93^^,^^95^^,^^102^^,^^105^^,^  ^106^ Choosing suitable metrics for model evaluation is crucial, as it can affect decisions regarding the technology usefulness. Overly good performance metrics may indicate bias or overfitting. Studies that did not clearly demonstrate adequate validation methodologies and/or train/test dataset independence may have overestimated performance.^14^^,^^17^^,^^18^^,^^20^^,^^23^^,^^25^^,^^26^^,^^30^^,^^31^^,^^40^^,^^42^^,^^45^^,^^55^^,^^56^^,^^62^^,^^63^^,^^66–69^^,^^75^^,^^76^^,^  ^78^^,^^79^^,^^81^^,^^82^^,^^87^^,^^90^^,^^91^^,^^99^^,^^101^^,^^102^^,^^105^ The monitoring of training and validation error rates ensures optimal model performance and prevents overfitting. Several studies used EV to assess performance.^11^^,^^15^^,^^23^^,^^27^^,^^32^^,^^46^^,^^49^^,^^54^^,^^57^^,^^65^^,^^70^^,^^86^^,^^96^^,^^106^ EV is the most demanding test of a model’s performance and a critical requirement before clinical deployment.^115^ Studies reported decreased performance with EV in 75% of cases,^11^^,^^15^^,^^27^^,^^28^^,^^32^^,^^46^^,^^57^^,^^65^^,^^70^^,^^86^^,^^96^^,^^106^ similar to findings in a systematic review on EV of DL algorithms for radiologic diagnosis.^116^

### Model explainability and clinical implications

The demand for AI expertise in the osteoporosis field is largely led by the current limited understanding and explanation of bone fragility and fracture. Improvements in the clinical workflow and in accuracy of practitioners were reported for fracture detection and classification applications,^63^^,^^64^ with performance comparable to experts for both fracture detection/classification and bone segmentation tasks.^64^^,^^65^^,^^69^^,^^70^^,^^98^^,^^100–102^ Demystifying algorithm decision-making plays a pivotal role in clinical acceptance. Most studies (64%) made efforts to adopt XAI strategies including activation maps for image-based applications in bone properties,^10^^,^^12^^,^^15^ osteoporosis classification,^27^^,^^28^^,^^41^ fracture detection,^46^^,^^47^^,^^57–60^^,^^62^^,^^63^ or risk prediction tasks.^85^ Other studies provided visualization of complex high-dimensional relationships between variables using semantic connectivity maps,^94^^,^^95^ SHAP,^17^^,^^34^^,^^83^^,^^92^ LIME,^40^ LASSO,^9^^,^^26^^,^^40^^,^^82^ feature importance engineering,^20^^,^^22^^,^^23^^,^^30^^,^^45^^,^^73–77^^,^^80^^,^^84^^,^^88^^,^^90^ sensitivity analysis,^10^^,^^49^^,^^50^^,^^54^^,^^69^^,^^89^ or error and thresholding analysis.^30^^,^^52^^,^^68^^,^^82^ Some studies deployed models as online applications, providing a platform for researchers and clinicians to access and expand validation beyond the original study.^77^^,^^82^^,^^83^^,^^89^

### Strengths and limitations of this study

The current work complements the report from Smets et al. by examining AI applications in osteoporosis after December 2020. This review discusses recent AI advances in osteoporosis and their clinical implications, showcasing novel screening and predictive techniques. Bone segmentation was introduced as a relevant AI domain. A thorough quality assessment was performed for each included study. However, there are several limitations to this review. First, our search was limited to the PubMed database, which may have resulted in the omission of relevant studies. Second, some studies overlapped between several application areas. The current work assigned a study to a given area based on the main AI output, regardless of its derived applications. Third, the quality assessment of the studies was done by only one assessor. Finally, the best performances are reported in Tables 1–5 with correlation coefficients (R2), AUC, accuracy or Dice when available, but these metrics may not be optimal for a given clinical scenario. Importantly, models should be assessed using multiple criteria.

## Conclusion

The field of osteoporosis management has witnessed a surge of interest in the application of AI, fueled by the emergence of big data, advancements in computing power, and increased accessibility to AI technologies. This review offers a comprehensive overview of recent AI developments in osteoporosis, and details some key aspects of model development, optimization, and reporting. A wide range of models and examination strategies have shown promise and warrant further EV. By providing interpretable explanations, AI models can bridge the gap between complex algorithms and clinical practice, facilitating their integration into routine healthcare workflows. However, the pathway to clinical implementation of AI models necessitates careful consideration of potential biases in the training data and model development. Establishing reporting standards that ensure high-quality AI research is imperative and will build confidence in AI-based approaches, ultimately leading to improved patient outcomes in osteoporosis management.

## Supplementary Material

Review_AI_SupplementaryMaterial_20240717_GATINEAU_et_al_zjae131

## Data Availability

The data supporting the findings of this study are available within the article and its supplementary materials. R and Python scripts were used to generate Figures 1 and 2, and are available from the corresponding author upon request.
