# Supplementary material for: Development and reporting of artificial intelligence in osteoporosis management
Source: J Bone Miner Res. 2024 Aug 20;39(11):1553–73. doi: 10.1093/jbmr/zjae131 (PMC11523092; doi:10.1093/jbmr/zjae131)
Supplement: Review_AI_SupplementaryMaterial_20240717_GATINEAU_et_al_zjae131 [file review_ai_supplementarymaterial_20240717_gatineau_et_al_zjae131.docx]

Supplementary materials

# PUBMED DATABASE SEARCH SYNTAX

The PubMed research syntax used was:

*("Osteoporosis"[Mesh] OR "Fractures, Bone"[Mesh:noexp] OR "Osteoporotic Fractures"[Mesh] OR "Hip Fractures"[Mesh] OR "Humeral Fractures"[Mesh] OR "Spinal Fractures"[Mesh] OR "Bone Density"[Mesh] OR Osteoporo*[tiab] OR "fragility fracture*"[tiab] OR (Fracture*[tiab] AND (humer*[tiab] OR spin*[tiab] OR vertebra*[tiab] OR hip[tiab] OR forearm[tiab])) OR "bone densit*"[tiab] OR "bone mineral densit*"[tiab]) AND ("Artificial Intelligence"[Mesh:noexp] OR "machine learning"[Mesh] OR "Neural Networks, Computer"[Mesh] OR "artificial Intelligence"[tiab] OR "machine learning"[tiab] OR "deep learning"[tiab] OR "neural network*"[tiab]) AND (2015[dp] :3000[dp]) AND English[la]*


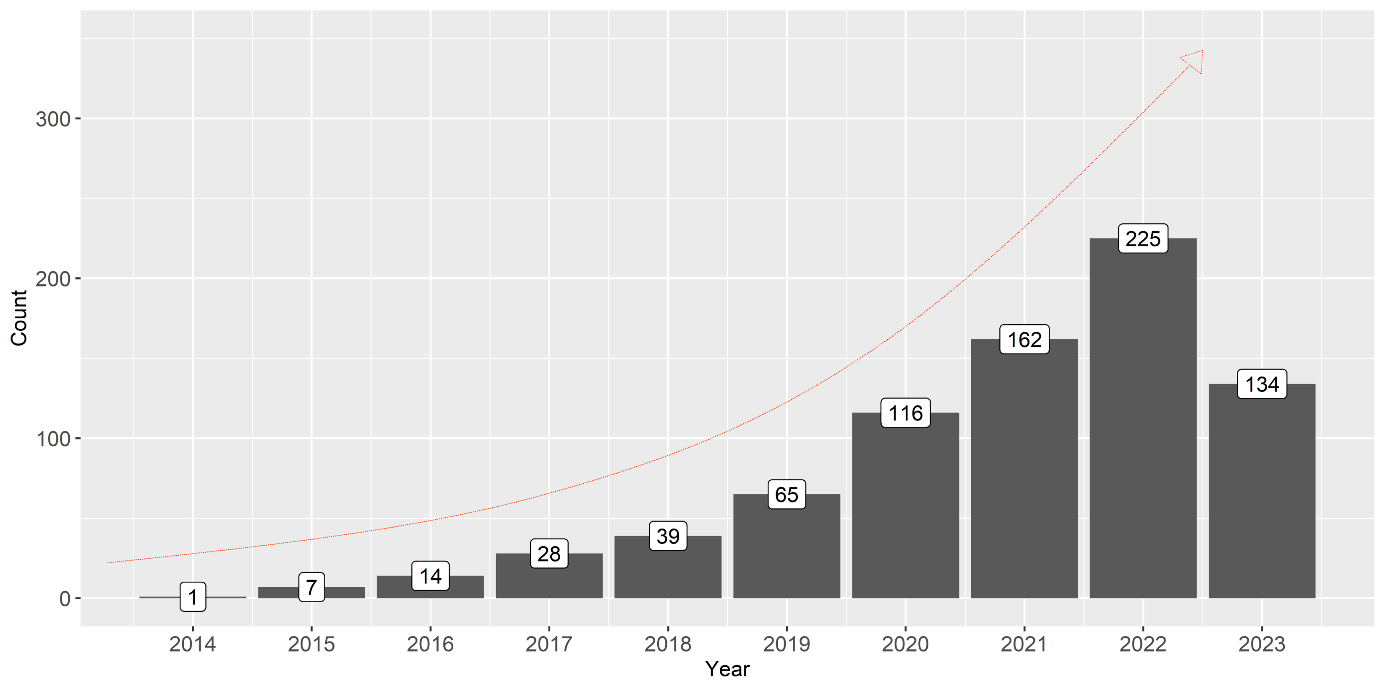


Figure 1: Results count per year of the syntax on PubMed search. These results have been generated in June 2023 (so year to mid-June for 2023) to get the latest tendency.

# QUALITATIVE ASSESSMENT

The Box 1 presents a simplified version of the MI-CLAIM checklist that was created from the 12 most relevant items to cover the 6 main quality assessment parts. Additional comments were provided to help the reader understanding key principles in AI development and reporting processes.

| **Study quality assessment (reporting and methodology)** | | |
| --- | --- | --- |
| **Study design (Part 1)** | **Completed:** | **Comments:** |
| The clinical problem in which the model will be employed is clearly detailed in the paper | ☐ | A well-defined research question or hypothesis outlining the problem that the AI system is designed to solve |
| The characteristics of the dataset (training and test sets if applicable) are detailed and have been shown to be representative to the research settings. | ☐ | Detailed description of the data characteristics, providing the information to which extend the data are representative of real-world data for the current research question/task. The inclusion of this information helps identify any biases or limitations of the study population or setting used, and determines AI system's performance alignment with real-world expectations and challenges |
| **Data and partitioning (Part 2)** | **Completed:** | **Comments:** |
| Transformations of the data before it is applied to the proposed model are described (e.g. missing data imputation, normalization, feature selection, etc.). | ☐ | Clear rationales for the necessity of the transformation, its impact on data improvement, and its contribution to the overall performance of the AI model should be stated. AI model´s input data transformations play a pivotal role in AI development, covering a wide range of techniques such as data cleaning, ROI selection, normalization, encoding, feature engineering, signal processing, and data augmentation. These techniques enhance the quality, relevance, and usability of the data while strengthening the overall predictive and generalization capabilities of the AI model. |
| The validation methodology is clearly explained. | ☐ | The validation methodology should include at least two different subsets of data: the training set and a test set. The training set constitutes the data from which the model will be developed, trained, and optimized; usually representing 60%-90% of the data. The test set represents the remaining “unseen” portion of data from which the model’s performance will be evaluated. Splitting the data in two different subsets allows training a model and evaluating it on unseen data. |
| The independence between training and test sets has been proven in the paper. | ☐ | No overlap should exist to prevent data leakage and artificially inflated model performance, which does not generalize well to new data. Transparent data splitting strategies and discussions ensure independence. Providing data characteristics for both sets identifies potential selection bias and class distributions. Using external cohorts or data as a test set is a reliable approach to prove independence, especially in medical applications. This involves using a separate dataset for evaluation, enabling an accurate assessment of performance. External cohorts assess model generalizability by including unseen devices or population characteristics. Incorporating external cohorts or data for testing provides a comprehensive evaluation and improves reliability. |
| **Model optimization and final model selection (Part 3)** | **Completed:** | **Comments:** |
| The state-of-the-art solution used as a baseline for comparison has been identified and detailed. | ☐ | State of the art solution that would serve as reference for external comparison. The baseline model should be a commonly used or easily interpretable model that provides a reference for performance comparison in the clinical setting. |
| Details on the models that were evaluated and the code developed to select the best model are provided. | ☐ | Full and clear description of each developed model with their hyperparameters should be provided. Reporting and justifying the model selection, its architecture, hyperparameters, and optimization strategy as based on the specific research task and data characteristics, ensures study reproducibility and transparency. The choice of model and parameters helps mitigate risks of overfitting and underfitting during training while optimizing performance. To optimize hyperparameters, grid search is a common method that involves training models with different hyperparameters combinations. Though computationally expensive, it identifies optimal model architectures and hyperparameter combinations. |
| **Model performance (Part 4)** | **Completed:** | **Comments:** |
| The primary metric selected to evaluate algorithm performance and clinical utility (e.g., AUC, F-score, etc.), including the justification for selection, has been clearly stated. | ☐ | Different tasks may require different metrics to accurately assess the effectiveness of a model. By leveraging proper evaluation metrics and understanding their concepts, developers and researchers can identify areas where the algorithm may need improvement and work towards enhancing its performance while making informed decisions towards medical usability. Details about classification, regression, segmentation tasks and the associated metrics for evaluation can be found in the [supplementary information sections 3 to 5](#_Classification_tasks), [Tables 1 to 3](#_SUPPLEMENTARY_TABLES) and [Figure 2](#_SUPPLEMENTARY_FIGURES). |
| The performance comparison between baseline and/or proposed model(s) is presented with the appropriate statistical significance. | ☐ | Results should be presented with at least confidence intervals (CI) or statistical significance allowing internal comparison. Comparing the performance of different machine learning models is important to determine which model is best suited for a particular task. However, looking at individual level performance metrics such as the accuracy or AUC-ROC is insufficient. Statistical comparisons or tests must be conducted to assess whether the differences in performance are significant. Further details are provided in the supplementary materials section 6. |
| **Model Examination (Part 5)** | **Completed:** | **Comments:** |
| An adequate examination technique has been reported (e.g. feature importance, sensitivity analysis, saliency maps, etc.) | ☐ | A model examination technique should be conducted to allow a clearer understanding of the model decision-making process and to ensure its reliability and safety for clinical decision-making processes. Key techniques include feature engineering, sensitivity analysis, and saliency maps. Feature engineering encloses a wide variety of statistical methods like the Least Absolute Shrinkage and Selection Operator (LASSO) or Principal Component Analysis (PCA) to identify influential predictors. Some more visual techniques like Shapley additive values (SHAP) or Local Interpretable Model-Agnostic Explanations (LIME) provide insights into both feature importance and contributions. Sensitivity analysis relies on exploring model robustness by examining input changes and predictions. Saliency maps are XAI techniques to visually explain important image regions for predictions. |
| A discussion of the reliability and robustness of the model as the underlying data distribution shifts is included. | ☐ | Objective discussion on strengths and weaknesses of the model to describe its effectiveness and trustworthiness in real-world setting. Reporting AI model´s strengths, limitations, and weaknesses helps to identify potential bias, making it easier to correct or mitigate them for further analyses. |
| **Reproducibility and transparency (Part 6)** | **Completed:** | **Comments:** |
| A link to the model development code or a framework to replicate the analysis have been provided. | ☐ | A documented open-source framework or source code should be provided to allow others to replicate the results of a study and verify the reliability of an AI system. |

Box 1: Simplified version of the MI-CLAIM checklist used for the qualitative assessment of the studies (based/modified from (7)). Comments sections were produced to help understanding the key principles of each item.

# INTRODUCTION TO AI SUBFIELDS AND MODEL SELECTION

Artificial Intelligence (AI) includes the subfields of Machine Learning (ML) and Deep Learning (DL), both of which can be trained using supervised learning, unsupervised learning, or reinforcement learning. Recent advancements in computing power have enabled the training of complex models and deep architectures from big data. The selection of appropriate ML/DL model depends on the research task, characteristics of the input data, and the desired outcome(s). ML/DL models are highly configurable with their own sets of hyperparameters that can be optimized for the specific task. Defining a proper model development and evaluation strategy is therefore crucial for identifying the best model configuration.

## Supervised Learning

In supervised learning, models are trained from labeled data (ground-truth) to make predictions or decisions based on input features. High quality ground-truth is crucial for effective learning and generalization.
Classical statistical models are:

- Linear Regression: Suited for regression tasks with few input features, continuous outcomes with linear relationships and low collinearity.
- Logistic Regression: Suited for binary classification tasks from numerical and categorical data, estimating probabilities of class membership.
- Cox Regression (proportional hazards regression): Suited for modeling time-to-event outcomes (survival analysis).

Classical ML models are:

- Decision Trees (DTs): Suited for both regression and classification tasks with non-linear relationships. DTs work with numerical and categorical input data.
- Random Forest (RF): It is an extension of DTs, suited for both classification and regression tasks. RF consists of training and aggregating, or bagging, multiple DTs with their own bootstrapped samples of data and randomized subsets of features. RF tends to improve the accuracy of a DT and reduce overfitting. Its architecture enables feature importance analysis strategies.
- Extreme Gradient Boosting (XGB): It is an extension of DTs, suited for both classification and regression tasks from numerical or categorical data. It relies on a boosting algorithm from multiple DTs and has a high performance and scalability. XGB architecture enables feature importance analysis.
- Support Vector Machines (SVM): It is suited for classification and regression tasks using numerical and/or encoded categorical data. The main concept underlying SVM is finding optimal decision boundaries in multidimensional space.
- Multi-layer Perceptron (MLP): MLPs are suited for both classification and regression tasks from numerical or categorical (encoded) variables. MLPs are a form of artificial neural networks (ANNs) which consist of an input layer (input features), potential intermediate layers and an output layer. MLPs are suitable for learning complex nonlinear relationships in data with large numbers of features.
- Convolutional Neural Networks (CNNs) are a specialized type of ANN, capable of capturing spatial hierarchies. CNNs are particularly effective for image/signal-based classification, regression, and segmentation tasks. These models are highly configurable in terms of architectures and hyperparameters. U-Net CNNs are specialized in segmentation tasks and rely on encoder-decoder networks that are connected by a bottleneck layer, creating a U-shaped architecture. U-Net CNNs enable the extraction of pixel-level relationships while retaining high-level image information, facilitating the reconstruction of a bone mask with dimensions like those of the original input image.
- Recurrent Neural Networks (RNN): RNNs are a variation in ANN models with dedicated memory allocation to process information over time. RNNs are used for regression and classification tasks from sequential or time-series data.

## Unsupervised Learning

Unsupervised learning attempts to discover hidden patterns or structures from unlabeled data. Common unsupervised learning models are:

- K-Means: K-means model works by partitioning numerical data into K-clusters based on similarity and Euclidean distance between data points. K-modes is an extension of K-means for categorical input data which uses different dissimilarity measures instead of Euclidean distance.
- Principal Component Analysis (PCA). PCA is useful for reducing the dimensionality to a limited number of factorial axes, reducing computational costs, and visualizing high-dimensional relationships.

# SUPPLEMENTARY INFORMATION

The supplementary information below discusses essential topics such as stratification techniques, cross-validation methodologies, and the fundamental concepts about classification, regression, and segmentation tasks. By exploring these supplementary paragraphs, readers will gain deeper insights into the foundational principles discussed in the main body of the article.

## Stratification technique

The stratification is a robust method which consists in splitting a dataset into a training set and a testing set with equally distributed classes of interest or ground truth. This can also be applied in a cross-validation strategy (see [2. Cross-validation below](#_Cross-validation)). In fact, if the training and testing sets have a different classes distributions, the model may not be able to generalize well to unseen data and will have high variance in its performance. Stratification is particularly important when the dataset is imbalanced, meaning that one class is much more common than the other. In imbalanced dataset scenario, randomly splitting it into a training set and a testing set may result in having very few samples of the minority class in one or the other split, leading to inaccurate evaluation of the model's performance.

## Cross-validation

In addition to the train and test split, a validation set can also be established from the training set to monitor the training process and fine-tune the model’s hyperparameters from intermediate evaluation steps. In this regards, a robust and commonly used validation strategy is the Leave One Out Cross-Validation (LOOCV), also named K-fold cross-validation as shown in the [supplementary Figure 1](#_SUPPLEMENTARY_FIGURES). This technique consists in splitting the training dataset in a number of $K$ disjoint folds, where $K - 1$ parts are used for training and the remaining used for evaluation (validation). This process is iteratively repeated $K$ times for all possible combinations, producing $K$ different test errors. The mean error and confidence intervals from all iterations shall provide insights on the model’s training process and generalization, to further fine-tune its architecture and hyperparameters and reach optimum performances.

## Classification tasks

Classification tasks consist in predicting the class or category of a given input. In other words, classification models learn to distinguish between different classes based on labeled training data. Commonly used metrics for classification tasks include accuracy (also known as positive predicted value or PPV), precision, recall (also known as sensitivity), specificity, negative predictive value (NPV), F1 score, area under the receiver operating characteristic curve (AUC-ROC), and area under the precision-recall curve (AUC-PR). The performance of a classification model should be evaluated according the medical context and the aim of the model, and motivated by researchers in this regard. As an example, it would be more important to prioritize high recall over high precision when evaluating a machine learning model in a life-threatening medical scenario, such as cancer detection. This is because recall measures the proportion of true positives that are correctly identified by the model, which is particularly important to capture all positive cases. On the other hand, high precision is important for tasks where false positives can have significant negative consequences such as unnecessary surgeries or heavy treatments. In any case, a thorough understanding of the research task and its clinical outcome(s) is necessary to train, optimize and assess the model performance accordingly. Another important performance evaluation tool for classification models is the confusion matrix. A confusion matrix is a table that summarizes the number of correct and incorrect predictions made by a classification model. This matrix provides a detailed view of the classifier's performance, and enables the computations of subsequent performance metrics. The confusion matrix is typically presented in a 2x2 table for binary classification tasks, with rows representing the actual class (ground truth) and columns representing the predicted class as shown in the [supplementary Figure 2](#_SUPPLEMENTARY_FIGURES). The four entries in such table represent the number of TP, FP, FN, and TN. By analyzing the values in a confusion matrix, we can gain insights into the strengths and weaknesses of a classifier.

## Regression tasks

Regression tasks evaluate the ability of a model to predict continuous numerical outcomes. Common regression metrics include the Mean Squared Error (MSE), which measures the average squared difference between the predicted values (noted $ŷ_{i}$) and the actual values (ground truth, noted $y_{i}$) over n observations, where n is the total number of observations. The Mean Absolute Error (MAE) is another commonly used regression metric that measures the average absolute difference between the predicted values ($ŷ_{i}$) and the actual values ($y_{i}$) over n observations. R-squared ($R^{2}$) is a metric that represents the proportion of variance in the dependent variable ($y$) that can be explained by the independent variables ($ŷ$), and ranges from 0 to 1. Calibration, or calibration slope and intercept, is another metric that evaluates how closely the predicted values match the actual values by examining the relationship between the predicted values ($ŷ_{i}$) and the average actual value ($ȳ$).

## Segmentation tasks

Segmentation tasks are specifically designed to divide an image into meaningful segments or regions, with each segment representing a distinct object or area of interest. In this context, the performance of AI models is assessed using metrics that evaluate the alignment or overlap between the predicted segmentation and the ground truth. This overlap is typically quantified using various statistical measures, such as the Intersection over Union (IoU) or the Dice coefficient. These metrics determine the degree of agreement between the predicted segmentation and the ground truth, allowing for an objective assessment of the algorithm's performance. In the context of segmentation tasks, the TP refers to pixels correctly classified by the algorithm as belonging to the target class. It indicates the algorithm's ability to accurately identify and assign the correct label to the pixels within the target region. On the other hand, the FP refers to the pixels that are incorrectly classified by the algorithm as belonging to the target class. It represents pixels that have been erroneously included within the target segment.

## Comparison of different models performance with appropriate statistics

Diverse techniques allow the comparison of models performances. A commonly used non-parametric statistical test to compare two AUC-ROC curves is the DeLong test. The DeLong test compares the AUC-ROC of two models by comparing the differences in ranks of the predicted probabilities between the two models. Confidence intervals (CI) are other useful statistical tools for assessing the variability and global performance of a model. By providing a measure of uncertainty around the estimated model performance, CI can help to assess the reliability or variance of the model and provide insights into the potential range of outcomes. This information can be valuable in making informed decisions about model selection and in quantifying the level of confidence that can be placed in the model's performance. Bootstrapping strategies can be used as a robust approach to estimate the variability in the performance metrics by resampling the test set with replacement. With bootstrapping, the performance metrics can be computed for each resampled test set and CI can be provided for each model using their resampled distributions.

# *SUPPLEMENTARY TABLES*

| ***TASK*** | ***Metric*** | ***Formula*** | ***Purpose*** |
| --- | --- | --- | --- |
| ***Classification tasks*** | *Accuracy - PPV* | $\frac{TP+TN}{TP+TN+FP+FN}$ | *Percentage of correctly predicted labels among all the predictions* |
|  | *NPV* | $\frac{TN}{TN+FN}$ | *Proportion of true negative predictions out of the total negative predictions. Indicator of how well a model can correctly identify negative cases.* |
|  | *Precision* | $\frac{TP}{TP+FP}$ | *Proportion of correctly predicted positive instances out of the total instances predicted as positive. Precision is useful in scenarios where false positives have significant consequences.* |
|  | *Recall - Sensitivity* | $\frac{TP}{TP+FN}$ | *Proportion of correctly predicted positive instances out of all actual positive instances. It emphasizes the ability of a model to identify positive instances correctly. Recall (or sensitivity) is essential in situations where false negatives have significant consequences.* |
|  | *Specificity* | $\frac{TN}{FP+TN}$ | *Proportion of correctly predicted negative instances out of all actual negative instances. It highlights the ability of a model to identify negative instances correctly. Specificity is valuable when it is crucial to avoid false positives.* |
|  | *F1-Score* | $2*\frac{Precision*Recall}{Precision+Recall}$ | *The F1-Score combines precision and recall into a single metric. It provides a balanced measure by calculating the harmonic mean of precision and recall. F1-Score is useful when both false positives and false negatives need to be minimized simultaneously.* |
|  | *AUC-ROC* | *Area under the curve of the TP rate against FP rate* | *Commonly used for binary classification problems, AUC-ROC quantifies the model's ability to distinguish between positive and negative instances by plotting the true positive rate against the false positive rate. Commonly used metric to represent the overall model performance.* |
|  | *AUC-PR* | *Area under the curve of the Precision against Recall* | *The Area Under the Precision-Recall Curve (AUC-PR) is another metric used for binary classification tasks. It evaluates the trade-off between precision and recall at various probability thresholds. AUC-PR is particularly useful when dealing with imbalanced datasets where the positive class is rare or when the focus is on the performance of the positive class.* |

Supplementary Table 1: Evaluation metrics for classification tasks

| ***TASK*** | ***Metric*** | ***Formula*** | ***Purpose*** |
| --- | --- | --- | --- |
| ***Segmentation tasks*** | *Accuracy - PPV* | $\frac{TP+TN}{TP+TN+FP+FN}$ | *Percentage of correctly predicted labels among all the predictions* |
|  | *NPV* | $\frac{TN}{TN+FN}$ | *Proportion of true negative predictions out of the total negative predictions. Indicator of how well a model can correctly identify negative cases.* |
|  | *Precision* | $\frac{TP}{TP+FP}$ | *Proportion of correctly predicted positive instances out of the total instances predicted as positive. Precision is useful in scenarios where false positives have significant consequences.* |
|  | *Recall – Sensitivity* | $\frac{TP}{TP+FN}$ | *Proportion of correctly predicted positive instances out of all actual positive instances. It emphasizes the ability of a model to identify positive instances correctly. Recall (or sensitivity) is essential in situations where false negatives have significant consequences.* |
|  | *Specificity* | $\frac{TN}{FP+TN}$ | *Proportion of correctly predicted negative instances out of all actual negative instances. It highlights the ability of a model to identify negative instances correctly. Specificity is valuable when it is crucial to avoid false positives.* |
|  | *F1-Score* | $2*\frac{Precision*Recall}{Precision+Recall}$ | *The F1-Score combines precision and recall into a single metric. It provides a balanced measure by calculating the harmonic mean of precision and recall. F1-Score is useful when both false positives and false negatives need to be minimized simultaneously.* |
|  | *AUC-ROC* | *Area under the curve of the TP rate against FP rate* | *The Area Under the Receiver Operating Characteristic Curve (AUC-ROC) is commonly used for binary classification problems. It quantifies the model's ability to distinguish between positive and negative instances by plotting the true positive rate against the false positive rate. Commonly used metric to represent overall performance.* |
|  | *AUC-PR* | *Area under the curve of the Precision against Recall* | *The Area Under the Precision-Recall Curve (AUC-PR) is another metric used for binary classification tasks. It evaluates the trade-off between precision and recall at various probability thresholds. AUC-PR is particularly useful when dealing with imbalanced datasets where the positive class is rare or when the focus is on the performance of the positive class.* |
|  | *Intersection over Union (IoU) – Jaccard Index* | $\frac{TP}{TP+FP+FN}$ | *IoU measures the overlap between the predicted and true regions of interest in tasks such as object detection or image segmentation. It is calculated by dividing the intersection area by the union area of the two regions. IoU provides a measure of the spatial accuracy of the model's predictions.* |
|  | *Mean Intersection over Union (mIoU)* | $\frac{1}{n_{c}}\sum_{i=1}^{n_{c}} {IoU}_{c_{i}}$ | *mIoU is the average of IoU scores calculated for multiple classes or regions. It is widely used in tasks involving multiple object or instance segmentation. mIoU provides an overall measure of segmentation accuracy across different classes.* |
|  | *Dice Index* | $\frac{2*TP}{2*TP+FP+FN}$ | *The Dice Index is similar to IoU and measures the overlap between the predicted and true regions. Dice Index is advantageous when the dataset is highly imbalanced and less sensitive to class prevalence than IoU.* |

Supplementary Table 2: Evaluation metrics for segmentation tasks

| ***TASK*** | ***Metric*** | ***Formula*** | ***Purpose*** |  |
| --- | --- | --- | --- | --- |
| ***Regression tasks*** | *Mean Squared Error (MSE)* | $\frac{1}{n}\sum_{i=1}^{n} {{(y}_{i}-ŷ_{i})}^{2}$ | *MSE calculates the average squared difference between the predicted and true values. It is commonly used in regression tasks and provides a measure of the average magnitude of the model's errors. MSE penalizes larger errors more than smaller ones.* |  |
|  | *Mean Absolute Error (MAE)* | $\frac{1}{n}\sum_{i=1}^{n} \vert y_{i}-ŷ_{i}\vert$ | *MAE is another widely used metric for regression models. It calculates the average of the absolute differences between the predicted and true values. Unlike MSE, MAE does not square the differences, making it less sensitive to outliers. MAE provides a measure of the average absolute magnitude of the errors. It is useful when the focus is on the average magnitude of errors and outliers should not have a disproportionate influence on the evaluation.* |  |
|  | *Coefficient of determination (CD), R2* | $1-\frac{\sum_{i=1}^{n} {{(y}_{i}-ŷ_{i})}^{2}}{\sum_{i=1}^{n} {{(y}_{i}-ȳ)}^{2}}$ | *R2, or the coefficient of determination, is a metric commonly used in regression analysis. It measures the proportion of the variance in the dependent variable (target) that can be explained by the independent variables (features) used in the model. R^2 ranges from 0 to 1, with a higher value indicating a better fit of the model to the data. R^2 provides an indication of how well the model captures the underlying patterns and variability in the data. It is valuable for assessing the overall goodness of fit of a regression model.* |  |
|  |  |  |  |  |
|  | *Calibration* | *Plot of the predicted outcome against the ground truth* | *Calibration refers to the alignment between predicted probabilities or confidence scores and the true probability of an event occurring. In other terms, it allows to assess how closely the points lie against identity (45-degree line)* |  |

Supplementary Table 3: Evaluation metrics for regression tasks

| *Reference* | *Research task* | *Data characteristics* | *Data transformations* | *Validation methodology* | *Train/test independence* | *Use of baseline* | *Model configuration and parameters* | *Performance metric* | *Comparison with appropriate statistics* | *Examination technique* | *Reliability and robustness discussion* | *Reproducibility and transparency* | *Quality score* |
| --- | --- | --- | --- | --- | --- | --- | --- | --- | --- | --- | --- | --- | --- |
| *Dai et al.* | *1* | *1* | *0* | *1* | *1* | *1* | *1* | *1* | *1* | *0* | *1* | *0* | ***9*** |
| *Ho et al.* | *1* | *1* | *1* | *1* | *1* | *0* | *1* | *1* | *1* | *1* | *1* | *0* | ***10*** |
| *Hsieh et al.* | *1* | *1* | *1* | *1* | *1* | *1* | *1* | *1* | *1* | *0* | *1* | *1* | ***11*** |
| *Kang et al.* | *1* | *1* | *0* | *1* | *1* | *0* | *1* | *0* | *0* | *1* | *1* | *0* | ***7*** |
| *Min et al.* | *1* | *1* | *1* | *1* | *1* | *0* | *1* | *0* | *0* | *0* | *1* | *0* | ***7*** |
| *Nguyen et al.* | *1* | *1* | *1* | *1* | *0* | *1* | *1* | *1* | *1* | *0* | *1* | *0* | ***9*** |
| *Nissinen et al.* | *1* | *1* | *1* | *1* | *1* | *1* | *1* | *1* | *1* | *1* | *1* | *0* | ***11*** |
| *Sato et al.* | *1* | *1* | *0* | *1* | *1* | *0* | *0* | *1* | *1* | *0* | *1* | *0* | ***7*** |
| *Tanphiriyakun et al.* | *1* | *1* | *1* | *1* | *0* | *0* | *1* | *0* | *1* | *1* | *1* | *0* | ***8*** |
| *Xiao et al.* | *1* | *1* | *1* | *1* | *0* | *1* | *1* | *1* | *1* | *0* | *1* | *0* | ***9*** |
| *Zhang et al.* | *1* | *1* | *1* | *1* | *1* | *1* | *0* | *1* | *1* | *1* | *1* | *0* | ***10*** |

Supplementary table 4: Bone properties assessment studies quality scores

| *Reference* | *Research task* | *Data characteristics* | *Data transformations* | *Validation methodology* | | *Train/test independence* | *Use of baseline* | *Model configuration and parameters* | *Performance metric* | *Comparison with appropriate statistics* | *Examination technique* | *Reliability and robustness discussion* | | *Reproducibility and transparency* | | *Quality score* |
| --- | --- | --- | --- | --- | --- | --- | --- | --- | --- | --- | --- | --- | --- | --- | --- | --- |
| *Biamonte et al.* | *1* | *1* | *0* | *1* | *0* | | *0* | *0* | *0* | *1* | *1* | | *1* | *0* | ***6*** | |
| *Bui et al.* | *1* | *1* | *0* | *1* | *1* | | *1* | *1* | *0* | *1* | *1* | | *1* | *0* | ***9*** | |
| *Chen et al.* | *1* | *0* | *1* | *1* | *1* | | *1* | *1* | *1* | *0* | *1* | | *1* | *0* | ***9*** | |
| *Chen et al.* | *1* | *1* | *1* | *0* | *1* | | *0* | *0* | *0* | *1* | *1* | | *1* | *0* | ***7*** | |
| *Erjiang et al.* | *1* | *1* | *1* | *1* | *1* | | *1* | *0* | *1* | *1* | *0* | | *1* | *0* | ***9*** | |
| *Fasihi et al.* | *1* | *1* | *0* | *1* | *0* | | *0* | *1* | *0* | *0* | *0* | | *1* | *0* | ***5*** | |
| *Huang et al.* | *1* | *1* | *1* | *1* | *0* | | *0* | *1* | *1* | *0* | *1* | | *1* | *0* | ***8*** | |
| *Jang et al.* | *1* | *0* | *1* | *1* | *1* | | *0* | *0* | *1* | *1* | *1* | | *1* | *0* | ***8*** | |
| *Jang et al.* | *1* | *1* | *1* | *1* | *1* | | *0* | *1* | *1* | *1* | *1* | | *1* | *0* | ***10*** | |
| *Kwon et al.* | *1* | *1* | *1* | *1* | *1* | | *0* | *0* | *1* | *1* | *1* | | *0* | *0* | ***8*** | |
| *Liu et al.* | *1* | *1* | *1* | *1* | *0* | | *0* | *0* | *0* | *1* | *1* | | *1* | *0* | ***7*** | |
| *Luo et al.* | *1* | *1* | *1* | *1* | *0* | | *1* | *1* | *1* | *1* | *0* | | *1* | *0* | ***9*** | |
| *Mao et al.* | *1* | *1* | *1* | *1* | *1* | | *1* | *1* | *1* | *1* | *0* | | *1* | *0* | ***10*** | |
| *Ou Yang et al.* | *1* | *1* | *0* | *1* | *0* | | *1* | *1* | *1* | *1* | *0* | | *1* | *0* | ***8*** | |
| *Park et al.* | *1* | *1* | *1* | *1* | *1* | | *1* | *0* | *1* | *1* | *1* | | *1* | *0* | ***10*** | |
| *Sebro et al.* | *1* | *1* | *0* | *1* | *1* | | *0* | *1* | *0* | *1* | *0* | | *1* | *0* | ***7*** | |
| *Sebro et al.* | *1* | *1* | *0* | *1* | *1* | | *0* | *1* | *0* | *1* | *0* | | *1* | *0* | ***7*** | |
| *Sebro et al.* | *1* | *1* | *0* | *1* | *1* | | *0* | *1* | *0* | *1* | *0* | | *1* | *0* | ***7*** | |
| *Sebro et al.* | *1* | *1* | *0* | *1* | *1* | | *0* | *1* | *0* | *1* | *0* | | *1* | *0* | ***7*** | |
| *Shen et al.* | *1* | *1* | *0* | *1* | *1* | | *0* | *0* | *0* | *1* | *1* | | *1* | *0* | ***7*** | |
| *Suh et al.* | *1* | *1* | *0* | *0* | *1* | | *1* | *0* | *0* | *1* | *1* | | *1* | *0* | ***7*** | |
| *Sukegawa et al.* | *1* | *1* | *1* | *1* | *1* | | *1* | *1* | *1* | *1* | *1* | | *1* | *0* | ***11*** | |
| *Wang et al.* | *1* | *1* | *1* | *1* | *0* | | *0* | *0* | *0* | *1* | *0* | | *1* | *0* | ***6*** | |
| *Widyaningrum et al.* | *1* | *1* | *1* | *1* | *1* | | *0* | *0* | *0* | *0* | *0* | | *1* | *0* | ***6*** | |
| *Yamamoto et al.* | *1* | *1* | *1* | *1* | *1* | | *1* | *1* | *0* | *1* | *0* | | *1* | *0* | ***9*** | |
| *Yang et al.* | *1* | *1* | *0* | *0* | *0* | | *0* | *0* | *0* | *1* | *1* | | *1* | *0* | ***5*** | |

Supplementary table 5: Osteoporosis classification studies quality scores

| *Reference* | *Research task* | *Data characteristics* | *Data transformations* | *Validation methodology* | *Train/test independence* | *Use of baseline* | *Model configuration and parameters* | *Performance metric* | *Comparison with appropriate statistics* | *Examination technique* | *Reliability and robustness discussion* | *Reproducibility and transparency* | *Quality score* |
| --- | --- | --- | --- | --- | --- | --- | --- | --- | --- | --- | --- | --- | --- |
| *Bae et al.* | *1* | *1* | *0* | *1* | *1* | *1* | *0* | *1* | *1* | *1* | *1* | *0* | ***9*** |
| *Chen et al.* | *1* | *1* | *1* | *1* | *1* | *0* | *1* | *1* | *1* | *1* | *1* | *1* | ***11*** |
| *Cheng et al.* | *1* | *1* | *0* | *1* | *1* | *1* | *1* | *0* | *1* | *1* | *1* | *1* | ***10*** |
| *Chou et al.* | *1* | *1* | *0* | *1* | *1* | *1* | *0* | *0* | *1* | *1* | *1* | *0* | ***8*** |
| *Del Lama et al.* | *1* | *0* | *1* | *1* | *1* | *1* | *0* | *0* | *1* | *1* | *1* | *1* | ***9*** |
| *Dong et al.* | *1* | *1* | *1* | *1* | *1* | *0* | *1* | *1* | *1* | *1* | *1* | *0* | ***10*** |
| *Germann et al.* | *1* | *1* | *1* | *1* | *1* | *1* | *1* | *0* | *1* | *1* | *1* | *0* | ***10*** |
| *Guermazi et al.* | *1* | *1* | *0* | *1* | *1* | *1* | *0* | *1* | *1* | *1* | *1* | *0* | ***9*** |
| *Inoue et al.* | *1* | *1* | *0* | *1* | *0* | *1* | *1* | *0* | *1* | *0* | *1* | *0* | ***7*** |
| *Li et al.* | *1* | *1* | *0* | *1* | *0* | *1* | *1* | *0* | *0* | *1* | *1* | *0* | ***7*** |
| *Li et al.* | *1* | *1* | *0* | *1* | *1* | *1* | *0* | *0* | *1* | *1* | *1* | *0* | ***8*** |
| *Monchka et al.* | *1* | *1* | *0* | *1* | *1* | *1* | *1* | *0* | *0* | *1* | *1* | *0* | ***8*** |
| *Monchka et al.* | *1* | *1* | *1* | *1* | *1* | *1* | *1* | *0* | *1* | *1* | *1* | *0* | ***10*** |
| *Murphy et al.* | *1* | *1* | *1* | *1* | *1* | *1* | *0* | *1* | *1* | *1* | *1* | *0* | ***10*** |
| *Ozkaya et al.* | *1* | *0* | *0* | *1* | *1* | *1* | *0* | *0* | *1* | *0* | *0* | *0* | ***5*** |
| *Rosenberg et al.* | *1* | *0* | *1* | *1* | *0* | *0* | *1* | *1* | *0* | *1* | *1* | *0* | ***7*** |
| *Sato et al.* | *1* | *1* | *1* | *1* | *0* | *1* | *1* | *0* | *1* | *1* | *1* | *0* | ***9*** |
| *Twinpray et al.* | *1* | *0* | *1* | *1* | *1* | *1* | *0* | *1* | *1* | *0* | *1* | *0* | ***8*** |
| *Xu et al.* | *1* | *1* | *1* | *1* | *1* | *1* | *1* | *1* | *1* | *0* | *1* | *1* | ***11*** |
| *Yabu et al.* | *1* | *1* | *0* | *1* | *0* | *1* | *0* | *0* | *0* | *0* | *1* | *0* | ***5*** |
| *Yadav et al.* | *1* | *0* | *1* | *1* | *0* | *1* | *1* | *1* | *0* | *1* | *0* | *0* | ***7*** |
| *Yeh et al.* | *1* | *1* | *1* | *1* | *0* | *0* | *1* | *1* | *1* | *1* | *1* | *0* | ***9*** |
| *Yoda et al.* | *1* | *1* | *1* | *1* | *0* | *1* | *0* | *0* | *1* | *1* | *1* | *0* | ***8*** |
| *Zakharov et al.* | *1* | *0* | *1* | *1* | *1* | *0* | *1* | *1* | *1* | *0* | *1* | *0* | ***8*** |
| *Zhang et al.* | *1* | *1* | *1* | *1* | *1* | *0* | *0* | *0* | *1* | *0* | *0* | *0* | ***6*** |

Supplementary table 6: Fracture detection/classification studies quality scores

| *Reference* | *Research task* | *Data characteristics* | *Data transformations* | *Validation methodology* | *Train/test independence* | *Use of baseline* | *Model configuration and parameters* | *Performance metric* | *Comparison with appropriate statistics* | *Examination technique* | *Reliability and robustness discussion* | *Reproducibility and transparency* | *Quality score* |
| --- | --- | --- | --- | --- | --- | --- | --- | --- | --- | --- | --- | --- | --- |
| *Cary et al.* | *1* | *1* | *1* | *1* | *0* | *0* | *1* | *1* | *1* | *0* | *1* | *0* | ***8*** |
| *Chen et al.* | *1* | *1* | *1* | *1* | *1* | *1* | *1* | *0* | *0* | *1* | *1* | *0* | ***9*** |
| *Chen et al.* | *1* | *1* | *0* | *1* | *1* | *1* | *0* | *1* | *1* | *1* | *1* | *0* | ***9*** |
| *Cheng et al.* | *1* | *1* | *1* | *1* | *0* | *0* | *0* | *1* | *0* | *1* | *1* | *0* | ***7*** |
| *Coco Martin et al.* | *1* | *1* | *0* | *1* | *0* | *0* | *0* | *1* | *1* | *1* | *1* | *0* | ***7*** |
| *De Vries et al.* | *1* | *1* | *1* | *1* | *0* | *1* | *1* | *1* | *1* | *1* | *1* | *1* | ***11*** |
| *DeBaun et al.* | *1* | *1* | *0* | *0* | *0* | *0* | *0* | *0* | *1* | *0* | *1* | *0* | ***4*** |
| *Du et al.* | *1* | *0* | *1* | *1* | *0* | *0* | *1* | *0* | *1* | *0* | *0* | *0* | ***5*** |
| *Forssten et al.* | *1* | *1* | *1* | *1* | *1* | *1* | *1* | *1* | *1* | *1* | *1* | *0* | ***11*** |
| *Galassi et al.* | *1* | *1* | *1* | *1* | *0* | *0* | *0* | *0* | *1* | *0* | *1* | *0* | ***6*** |
| *Harris et al.* | *1* | *1* | *1* | *1* | *0* | *0* | *0* | *1* | *1* | *1* | *1* | *0* | ***8*** |
| *Kitcharanant et al.* | *1* | *1* | *1* | *1* | *1* | *0* | *1* | *1* | *1* | *1* | *1* | *0* | ***10*** |
| *Klemt et al.* | *1* | *1* | *0* | *1* | *0* | *0* | *0* | *0* | *1* | *1* | *1* | *0* | ***6*** |
| *Kong et al.* | *1* | *1* | *1* | *1* | *1* | *1* | *1* | *1* | *1* | *1* | *1* | *0* | ***11*** |
| *Lei et al.* | *1* | *1* | *0* | *1* | *1* | *0* | *0* | *1* | *1* | *0* | *1* | *0* | ***7*** |
| *Lu et al.* | *1* | *1* | *0* | *1* | *0* | *1* | *0* | *0* | *1* | *1* | *1* | *0* | ***7*** |
| *Ma et al.* | *1* | *1* | *0* | *1* | *1* | *1* | *0* | *1* | *1* | *1* | *1* | *0* | ***9*** |
| *Oosterhoff et al.* | *1* | *1* | *1* | *1* | *1* | *0* | *0* | *1* | *1* | *1* | *1* | *0* | ***9*** |
| *Poullain et al.* | *1* | *1* | *0* | *0* | *0* | *0* | *0* | *0* | *1* | *1* | *1* | *0* | ***5*** |
| *Shimizu et al.* | *1* | *1* | *0* | *0* | *1* | *0* | *0* | *0* | *1* | *0* | *0* | *0* | ***4*** |
| *Shtar et al.* | *1* | *1* | *1* | *1* | *0* | *1* | *1* | *1* | *1* | *1* | *1* | *0* | ***10*** |
| *Takahashi et al.* | *1* | *1* | *1* | *1* | *1* | *0* | *1* | *0* | *1* | *1* | *1* | *0* | ***9*** |
| *Ulivieri et al.* | *1* | *1* | *1* | *0* | *0* | *0* | *0* | *0* | *1* | *1* | *0* | *0* | ***5*** |
| *Ulivieri et al.* | *1* | *1* | *1* | *1* | *0* | *0* | *0* | *0* | *0* | *1* | *1* | *0* | ***6*** |

Supplementary table 7: Risk prediction studies quality scores

| ***Reference*** | ***Research task*** | ***Data characteristics*** | ***Data transformations*** | ***Validation methodology*** | ***Train/test independence*** | ***Use of baseline*** | ***Model configuration and parameters*** | ***Performance metric*** | ***Comparison with appropriate statistics*** | ***Examination technique*** | ***Reliability and robustness discussion*** | ***Reproducibility and transparency*** | ***Quality score*** |
| --- | --- | --- | --- | --- | --- | --- | --- | --- | --- | --- | --- | --- | --- |
| *Cheng et al.* | *1* | *1* | *1* | *1* | *1* | *0* | *1* | *1* | *1* | *1* | *1* | *0* | ***10*** |
| *Deng et al.* | *1* | *0* | *1* | *1* | *1* | *1* | *1* | *1* | *1* | *1* | *1* | *0* | ***10*** |
| *Kim et al.* | *1* | *0* | *1* | *1* | *1* | *1* | *1* | *1* | *1* | *1* | *1* | *0* | ***10*** |
| *Kim et al.* | *1* | *0* | *0* | *1* | *0* | *1* | *1* | *1* | *1* | *0* | *1* | *0* | ***7*** |
| *Park et al.* | *1* | *1* | *1* | *1* | *1* | *1* | *1* | *1* | *1* | *1* | *1* | *0* | ***11*** |
| *Suri et al.* | *1* | *1* | *1* | *1* | *0* | *1* | *1* | *1* | *1* | *1* | *1* | *0* | ***10*** |
| *Wang et al.* | *1* | *1* | *0* | *1* | *0* | *1* | *1* | *0* | *1* | *1* | *1* | *0* | ***8*** |
| *Wei et al.* | *1* | *1* | *1* | *1* | *1* | *1* | *1* | *1* | *1* | *1* | *0* | *0* | ***10*** |
| *Yang et al.* | *1* | *1* | *1* | *1* | *1* | *1* | *1* | *1* | *1* | *0* | *1* | *0* | ***10*** |
| *Yang et al.* | *1* | *1* | *1* | *1* | *0* | *0* | *1* | *0* | *0* | *0* | *1* | *0* | ***6*** |
| *Zhao et al.* | *1* | *1* | *0* | *1* | *1* | *0* | *1* | *0* | *0* | *1* | *1* | *0* | ***7*** |

Supplementary table 8: Bone segmentation studies quality scores

# *SUPPLEMENTARY FIGURES*

*
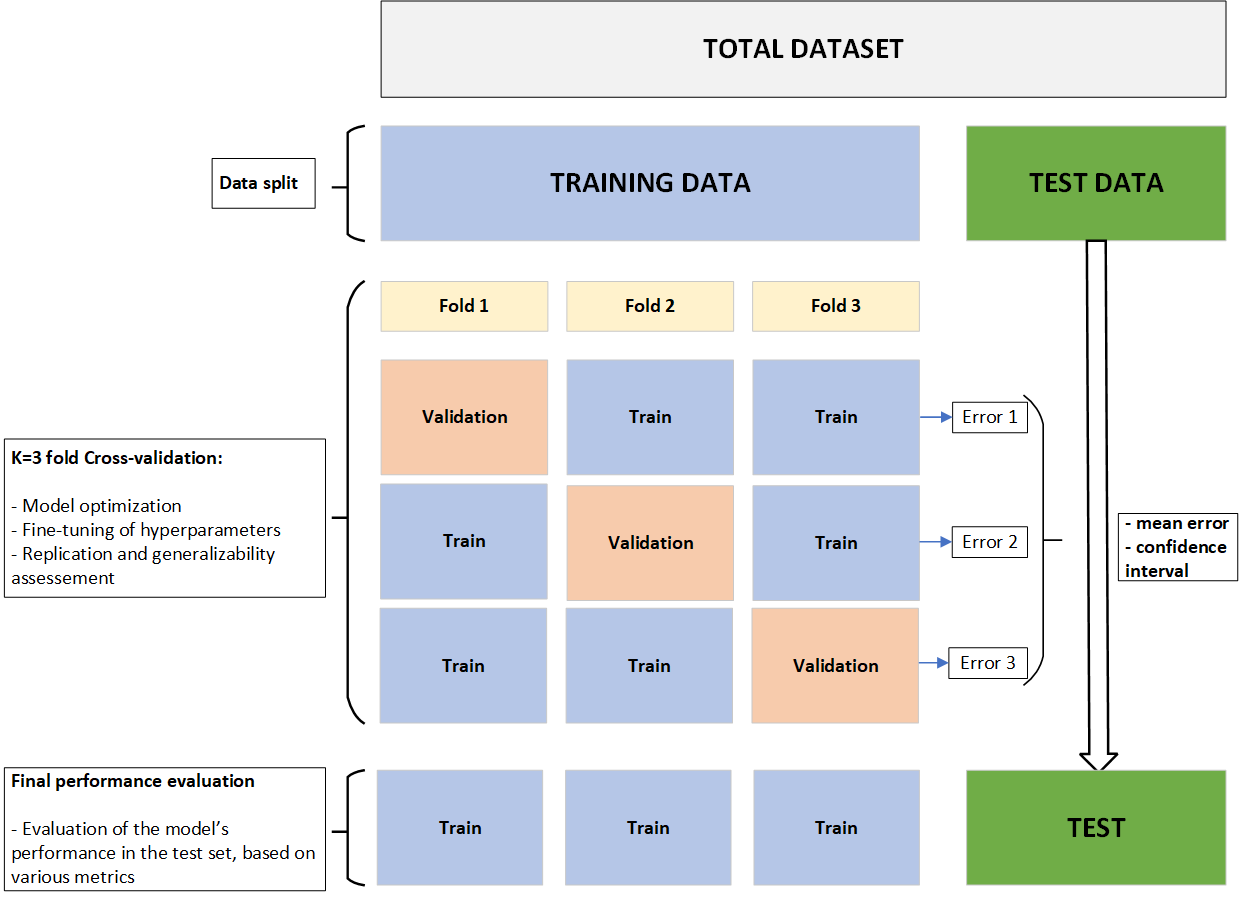
*

Supplementary Figure 1: k-fold cross validation example, with k=3

|  | *Predicted Positive (1)* | *Predicted  Negative (0)* |  |  |  |  |
| --- | --- | --- | --- | --- | --- | --- |
| *Actual Positive (1)* | *n(TP)* | *n(FN)* | *TOTAL actual positives* | |  | *correctly classified* |
| *Actual Negative (0)* | *n(FP)* | *n(TN)* | *TOTAL actual negatives* | |  | *misclassified* |
|  | *TOTAL predicted positives* | *TOTAL  predicted negatives* |  |  |  |  |
|  |  |  |  |  |  |  |

Supplementary Figure 2: Confusion Matrix of a binary classification task
